# Retrograde axonal transport of rabies virus is unaffected by interferon treatment but blocked by emetine locally in axons

**DOI:** 10.1371/journal.ppat.1007188

**Published:** 2018-07-20

**Authors:** Margaret A. MacGibeny, Orkide O. Koyuncu, Christoph Wirblich, Matthias J. Schnell, Lynn W. Enquist

**Affiliations:** 1 Department of Molecular Biology and Princeton Neuroscience Institute, Princeton University, Princeton, New Jersey, United States of America; 2 Department of Microbiology and Immunology, Thomas Jefferson University, Philadelphia, Pennsylvania, United States of America; Westmead Millennium Institute, AUSTRALIA

## Abstract

Neuroinvasive viruses, such as alpha herpesviruses (αHV) and rabies virus (RABV), initially infect peripheral tissues, followed by invasion of the innervating axon termini. Virus particles must undergo long distance retrograde axonal transport to reach the neuron cell bodies in the peripheral or central nervous system (PNS/CNS). How virus particles hijack the axonal transport machinery and how PNS axons respond to and regulate infection are questions of significant interest. To track individual virus particles, we constructed a recombinant RABV expressing a P-mCherry fusion protein, derived from the virulent CVS-N2c strain. We studied retrograde RABV transport in the presence or absence of interferons (IFN) or protein synthesis inhibitors, both of which were reported previously to restrict axonal transport of αHV particles. Using neurons from rodent superior cervical ganglia grown in tri-chambers, we showed that axonal exposure to type I or type II IFN did not alter retrograde axonal transport of RABV. However, exposure of axons to emetine, a translation elongation inhibitor, blocked axonal RABV transport by a mechanism that was not dependent on protein synthesis inhibition. The minority of RABV particles that still moved retrograde in axons in the presence of emetine, moved with slower velocities and traveled shorter distances. Emetine’s effect was specific to RABV, as transport of cellular vesicles was unchanged. These findings extend our understanding of how neuroinvasion is regulated in axons and point toward a role for emetine as an inhibitory modulator of RABV axonal transport.

## Author summary

Rabies virus (RABV) and alpha herpesviruses (αHV) (e.g. herpes simplex virus) evolved to enter the nervous system efficiently each time they infect a host. In most mammals, RABV reaches the brain, causing a fatal encephalitis. Whereas, αHV remain in the peripheral nervous system in a quiescent but reactivatable state. Despite distinct clinical outcomes, both RABV and αHV must invade axons and repurpose the axon transport machinery to travel long distances toward the neuronal cell bodies where virus replication occurs. How virus particles hijack the transport machinery and how axons respond to and regulate infection are questions of significant interest. We investigated how axonal RABV transport is regulated by exposing axons to interferons or protein synthesis inhibitors, both of which restrict transport of αHV particles. Unlike αHV infection, exposure of isolated axons to interferons has no effect on RABV neuroinvasion. However, RABV transport is blocked by axonal exposure to the translation elongation inhibitor, emetine, via a mechanism that does not depend on protein synthesis inhibition. The effect of emetine is not due to a global inhibition of axon transport because emetine does not limit axonal transport of cellular vesicles. Therefore, emetine may be a novel inhibitory modulator of RABV axonal transport.

## Introduction

Unlike most nervous system pathogens, which are either accidental or opportunistic, some neuroinvasive viruses have evolved strategies to enter and exit the nervous system. One successful strategy is that of rabies virus (RABV), a neuroinvasive human and animal pathogen of the *Rhabdoviridae* family. A zoonotic rabies infection begins with the bite of an infected animal into a muscle, followed by spread of virus particles through peripheral nervous system (PNS) somatic motor neurons and into the central nervous system (CNS). Spread of the infection from the CNS to salivary glands facilitates transmission to other hosts [1]. Nevertheless, the end result of CNS infection is a fatal encephalitis in humans and most mammals. A distinct but equally effective strategy is used by alpha herpesviruses (αHV) of the *Herpesviridae* family (e.g. human herpes simplex virus type 1 and 2 (HSV-1 and 2) and swine pseudorabies virus (PRV)) in their natural and non-natural hosts. These viruses replicate in peripheral epithelia prior to invading the innervating PNS neurons where they establish life-long latent infections. Unlike RABV, αHV rarely spread to the CNS in immunocompetent natural hosts. However, latent αHV infections undergo stress-induced reactivation, which can lead to peripheral herpetic lesions (e.g. cold sores) that facilitate inter-host spread. Despite their distinct clinical pathologies, both RABV and αHV must invade axons, something most viruses do not do. In addition, the infectious particles must travel long distances to reach the viral replication sites in the PNS/CNS cell bodies. How these distinct neuroinvasive viruses infect the nervous system efficiently remains a question of significant interest.

Due to the highly polarized nature of PNS neurons, axons function as molecular highways for long distance transport of various cellular cargos including proteins, RNAs, vesicles, and organelles [2]. Active transport between axon termini and cell bodies relies on microtubule-based molecular motors. In axons, cargos are transported away from the cell body, in the anterograde direction, by various microtubule plus-end directed kinesin motors. Cargos are transported toward the cell body, in the retrograde direction, by a single microtubule minus-end directed dynein motor that works in conjunction with dynein regulatory factors (e.g. dynactin, Lis1, and NudE/NudEL) [3,4]. For efficient axonal invasion, virus particles must co-opt the existing axonal transport machinery for long-distance retrograde transport, a process that has not been fully elucidated.

The axon is a neuronal sub-compartment capable of sensing external stimuli and mounting independent responses to local insults from the peripheral tissues including axon injury, infection, and exposure to trophic factors or pro-inflammatory cytokines [5–8]. Recently, it was shown that αHV re-purpose the retrograde axon injury signaling response for efficient virus particle transport in sympathetic superior cervical ganglia (SCG) axons. To do this, αHV infection induces rapid local translation of a subset of repressed axonal mRNAs without support from the connected cell bodies. Pharmacological inhibition of local protein synthesis restricts axonal αHV infection [7]. Furthermore, exposure of SCG axons to the pro-inflammatory cytokines, interferon-beta (IFNβ) or interferon-gamma (IFNγ), induced local antiviral responses in axons that limited the number of αHV particles transported in the retrograde direction [8]. These findings and others suggest that axons are independent sensors of peripheral αHV infection and raise the question if axons detect and respond to RABV infection in a similar manner.

RABV enters axons primarily by clathrin-dependent receptor-mediated endocytosis, which occurs when the viral envelope glycoprotein (G) binds to one of three known cell surface receptors: neural cell adhesion molecule (NCAM), p75 neurotrophin receptor (p75NTR), and n-acetylcholine receptor (nACHR) [9]. Viral particles in endosomes are transported retrograde until endosome acidification causes a conformational change in the G protein [10]. This change leads to fusion of the viral and endosome membranes and release of nucleocapsids into the cytosol. A majority of evidence suggests that RABV particles are released primarily in the cell body after long distance retrograde transport inside endosomes [11,12]. Although the RABV phosphoprotein (P) on the nucleocapsid was reported to bind directly to the LC8 dynein light chain [13,14], this interaction was not required for retrograde transport of RABV or CNS infection [15].

RABV, similar to αHV, might alter the axonal environment to re-purpose the axon transport machinery. Gluska et al. showed that RABV hijacks the neurotrophin signaling pathway in sensory dorsal root ganglia (DRG) axons by binding to and internalizing with the nerve growth factor (NGF) receptor, p75NTR [16]. Interestingly, RABV-containing endosomes moved retrograde with greater velocity and processivity than NGF-containing endosomes. However, the mechanism leading to faster retrograde transport of RABV-carrying vesicles is not well understood, and it is not clear whether pharmacological agents could act locally in PNS axons to regulate or block RABV transport. An additional complication was that previous studies were conducted with attenuated vaccine strains, which may have different transport properties than pathologic neurovirulent strains due to differences in their glycoproteins.

In this report, we extend the current understanding of RABV axonal invasion and transport by using a RABV P-mCherry-expressing recombinant derived from a neurovirulent strain (CVS-N2c) that is more neurotropic and less neurotoxic than the attenuated strains used previously [17]. Using a tri-chamber neuron culture system that physically separates axons from cell bodies, we show that RABV particles enter SCG axons and are transported efficiently in the retrograde direction. Unlike αHV infection, exposure of isolated axons to IFNβ or IFNγ has no effect on RABV neuroinvasion. Again, unlike axonal infection by αHV, RABV infection does not stimulate significant local protein synthesis. However, RABV transport is blocked by axonal exposure to the translation elongation inhibitor, emetine. Interestingly, emetine is the only protein synthesis inhibitor that blocked retrograde infection: Axonal treatment with cycloheximide or puromycin had no effect. The minority of RABV particles that do move in the retrograde direction in the presence of emetine, move with slower velocities and travel shorter distances. We find that the effect of emetine is not due to a global inhibition of axon transport because emetine does not limit the proportion of moving Rab5- or Rab7-positive vesicles in axons. Therefore, emetine must inhibit retrograde RABV transport by a mechanism that is independent of cytosolic protein synthesis inhibition, and it may be a novel inhibitory modulator of RABV axonal transport. In summary, these findings reveal that axons of PNS sympathetic neurons use distinct mechanisms to detect and respond to αHV and RABV.

## Results

### Construction of a CVS-N2c RABV recombinant for live-cell imaging in PNS neurons

**Fig 1:**
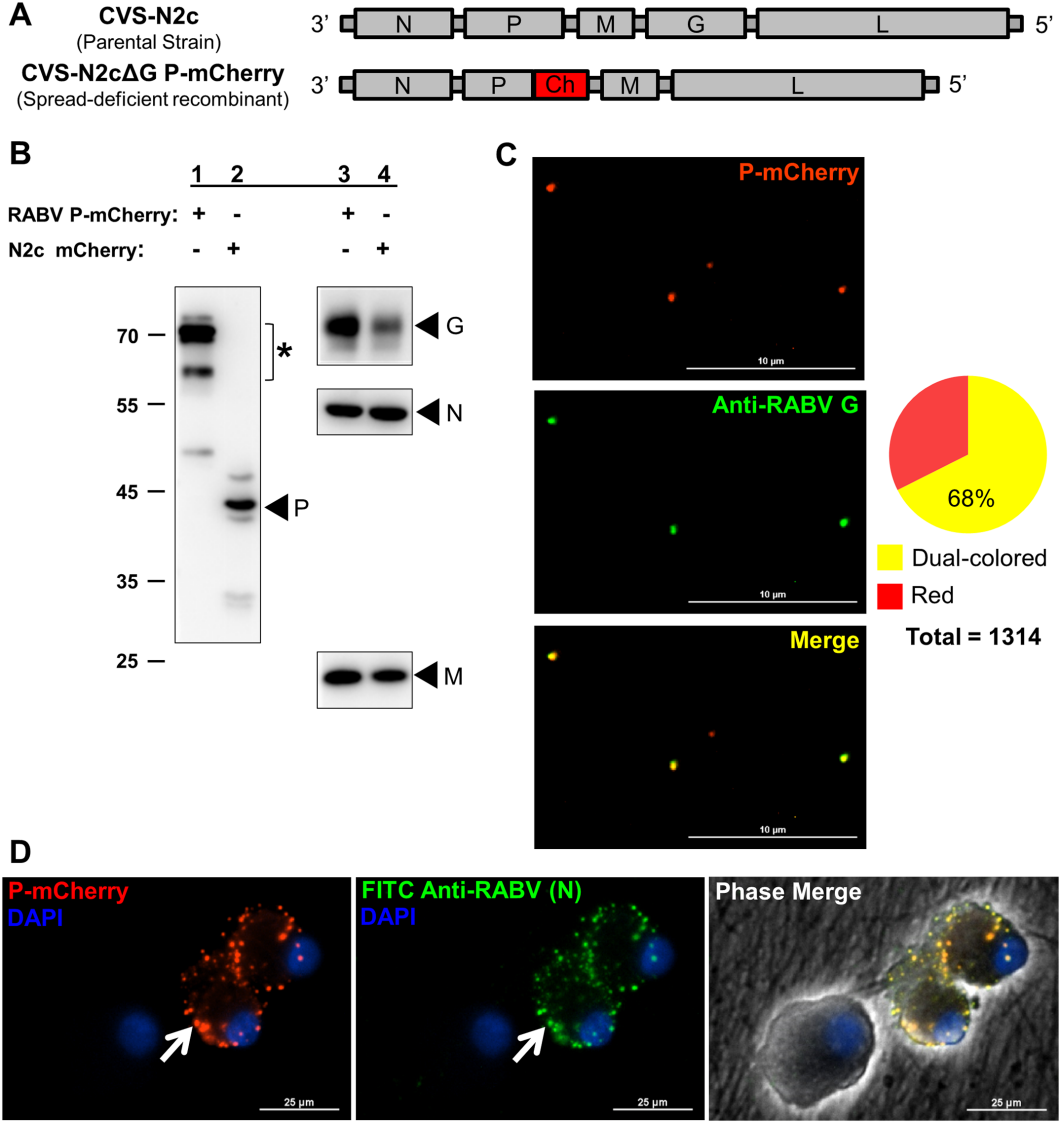
Construction of a CVS-N2c RABV recombinant for live-cell imaging in PNS neurons. **(A)** Genome schematics of the parental strain and P-mCherry-expressing, spread-deficient RABV recombinant. **(B)** Protein composition of sucrose-purified RABV P-mCherry particles (lanes 1 and 3) using SDS-PAGE and western blotting with anti-RABV P, G, N, or M. N2c mCherry particles (G-complemented N2cΔG expressing diffusible mCherry) are included for comparison (lanes 2 and 4). Asterisk indicates P-mCherry fusion protein. **(C)** Pie chart shows the proportion of enveloped virions (dual colored; 888) to non-enveloped nucleocapsids (red-only; 426) in the RABV P-mCherry stock (n = 1314 particles total). P-mCherry-positive particles (red) from purified supernatants were stained with anti-RABV G and Alexa Fluor 488-conjugated 2° antibody (green) (scale bars = 10 μm) (see also S1 Fig). **(D)** Dissociated SCG were infected with RABV P-mCherry (10^5^ ffu) (red) and stained at 48 hpi with FITC-conjugated anti-RABV targeting the N protein (green). DAPI stains nuclei (blue). White arrow indicates a cytoplasmic inclusion body (scale bar = 25μm).

RABV axon transport studies were conducted previously with attenuated vaccine strains (i.e. SAD B19) [18]. Attenuated strains display reduced neurotropism compared to virulent strains due to differences in the glycoprotein, which dictates the axon entry and retrograde transport properties of RABV [19,20]. To analyze neuroinvasion events by virulent RABV, we constructed a recombinant derived from the neuroinvasive CVS-N2c parental strain (Fig 1A) [21]. The recombinant expresses a RABV phosphoprotein-mCherry fusion protein (P-mCherry) that is incorporated into nucleocapsids to facilitate real-time visualization and tracking of single RABV particles. This P fusion was shown to be functional recently [22]. Prior to constructing the recombinant virus, the G gene was deleted from the N2c strain to create a spread-deficient virus (ΔG) that, when propagated on a G-complementing cell line, can infect a single round of cells but cannot spread to uninfected cells.

RABV(ΔG) P-mCherry was recovered from cloned cDNA that was transfected into Neuro2A mouse neuroblastoma (N2a) cells expressing the CVS-N2c G protein as described previously [23]. We refer to the G-complemented virus as RABV P-mCherry.

### Single RABV P-mCherry particles can be visualized by fluorescence microscopy

The protein composition of sucrose-purified RABV P-mCherry particles was verified using SDS-PAGE and western blotting with anti-RABV G, N, M, and P antibodies (Fig 1B). We next determined if sufficient P-mCherry protein was incorporated into nucleocapsids to visualize individual RABV particles by fluorescence microscopy. Diluted supernatants from RABV P-mCherry-infected, G-expressing N2a cells were spotted on glass coverslips. We observed red fluorescent punctae of relatively uniform shape from infected cell supernatants (Fig 1C, see also S1 Fig).

To determine the proportion of fully enveloped virions in the virus stock, particles on coverslips were stained with an antibody recognizing the RABV G protein on the envelope surface and green Alexa Fluor 488-conjugated secondary antibody. We observed green fluorescent punctae that were similar in shape to the red punctae in our virus stocks. 68% of the red and green punctae co-localized, suggesting that the majority of P-mCherry containing particles in the virus stock are intact, enveloped virions (Fig 1C).

We next determined the average number of P-mCherry protein copies per particle by comparing the particle brightness of RABV P-mCherry and a pseudorabies virus (PRV; swine αHV) recombinant expressing a capsid protein (pUL25)-mCherry fusion (S1A Fig). Because pUL25 is incorporated in precisely 60 copies in αHV capsids [24], we could extrapolate the P-mCherry protein copy number by comparing the fluorescence emission intensities between virus recombinants [25] (S1B Fig). RABV P-mCherry nucleocapsids contained approximately 116 copies of P-mCherry protein on average (S1C Fig). Thus, RABV nucleocapsids incorporate sufficient copies of P-mCherry protein for effective visualization of single virus particles.

### Sympathetic neurons are susceptible to RABV infection

We routinely model axonal invasion by neuroinvasive viruses using primary rodent sympathetic superior cervical ganglia (SCG) of the autonomic nervous system [26]. SCG ganglia contain a homogenous population of neurons that grow robust axons in the presence of NGF, which enables reproducible and well-controlled axonal infections [27]. However, to our knowledge, only a single study has investigated RABV infection in SCG *in vitro* [28]. Therefore, we first determined if SCG neurons are indeed susceptible to RABV infection. Dissociated SCG were infected with RABV P-mCherry and imaged for P-mCherry expression at 48 hours post infection (hpi). The P-mCherry signal was present in large punctae throughout the cytoplasm of dissociated SCG cell bodies (Fig 1D). Fixed cells were stained with FITC-conjugated anti-RABV targeting RABV nucleoprotein (N). We observed near complete co-localization between P-mCherry and N protein. Cytoplasmic inclusion bodies containing the RABV P and N proteins are characteristic of rabies replication compartments, suggesting that SCG neurons are susceptible and permissive to RABV infection *in vitro*.

### RABV infects sympathetic neurons in tri-chambers via axonal infection

The tri-chamber compartmented neuron culture system separates axons from cell bodies by two physical barriers. This system enables directional infections that mimic the natural route of nervous system invasion, where virus particles enter axons and undergo retrograde-directed transport toward distant cell bodies.

**Fig 2:**
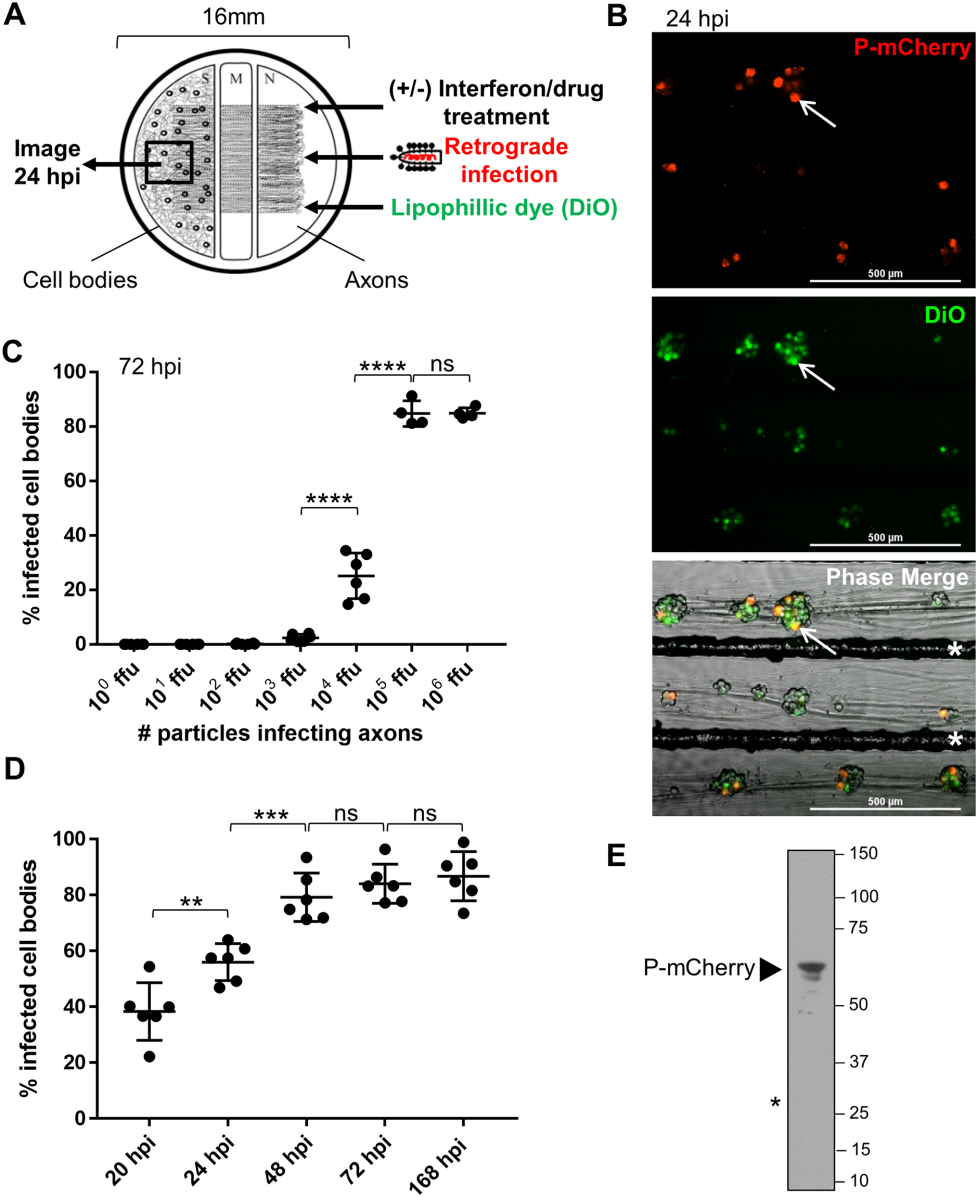
RABV infects sympathetic neurons in tri-chambers via axonal infection. **(A)** Retrograde infection in tri-chambers (S, soma (left); M, methocel (middle); N, neurite (right)). **(B)** Cell bodies in S at 24 h post RABV P-mCherry infection (10^5^ ffu) in N. White arrow indicates an infected cell body with axonal connection to N. White asterisks indicate etched grooves (scale bar = 500 μm). **(C)** Quantification of % infected cell bodies at 72 hpi when axons were infected with different numbers of viral particles (10^0^ ffu–10^6^ ffu). **(D)** Quantification of % infected cell bodies from 20 h to 168 h post axonal infection with 10^5^ ffu. Black dots in (C) and (D) represent individual tri-chambers (from three (C) and two (D) independent replicates). Horizontal lines and errors bars represent mean ± SD for each condition with **p = 0.01, ***p = 0.0006, ****p < 0.0001 using one-way ANOVA (ns = not significant). **(E)** Anti-mCherry western blot of protein lysate from S compartment cell bodies at 72 h post axonal infection (10^5^ ffu). Arrowhead indicates band for P-mCherry fusion protein (~60 kDa). Asterisk indicates expected size of unfused mCherry protein (~27 kDa), which is not present in infected cells.

SCG are grown in tri-chambers as described previously [29] (Fig 2A). Briefly, SCG cell bodies are dissociated and seeded in the S (soma) compartment. Cell bodies extend long axons that penetrate beneath the two barriers of the M (methocel/middle) compartment and into the N (neurite) compartment. To visualize and count the cell bodies that extend axons through to the N compartment, a green lipophilic dye (DiO) is added to the N compartment axons. This dye diffuses along axons to label the connected cell bodies in green (Fig 2A and 2B).

We first determined how many RABV particles must be added to axons to establish an infection in the cell bodies in the S compartment. We measured RABV infection based on viral protein expression in the cell bodies. Specifically, we used fluorescence microscopy to detect expression of the P-mCherry fusion protein. We infected axons with 10^0^, 10^1^, 10^2^, 10^3^, 10^4^, 10^5^, or 10^6^ focus forming units (ffu) of RABV P-mCherry and calculated the percentage of connected cell bodies that became infected based on P-mCherry expression. No P-mCherry expression was observed in the cell bodies when axons were infected with 10^0^ ffu. When axons were infected with 10^1^, 10^2^, and 10^3^ ffu, P-mCherry was expressed in a small percentage of the connected cell bodies (0.01 ± 0.03%, 0.2 ± 0.2%, and 2.4 ± 1.3%, respectively) (Fig 2C). When axons were infected with 10^4^, 10^5^, and 10^6^ ffu, P-mCherry was expressed in 25.2 ± 8.4%, 84.8 ± 4.7%, and 84.9 ± 2.0% of the connected cell bodies, respectively. In all conditions, the percentage of infected cell bodies did not increase after 72 hpi, confirming that the virus is spread-deficient. Therefore, a specific threshold of particles (≥ 10^4^ ffu) must be exceeded to establish an efficient retrograde RABV infection. The maximum cell body infection is reached when axons are infected with 10^5^ ffu, above which, the infection is saturated. All future axonal infection experiments were conducted using 10^5^ ffu of RABV P-mCherry.

To monitor the dynamics of retrograde RABV infection in SCG in tri-chambers, we calculated the percentage of infected cell bodies starting at 20 h and up to 168 h after axonal infection with 10^5^ ffu of RABV P-mCherry. The percent of cell bodies expressing P-mCherry increased significantly between 20 and 24 hpi (from 38.3 ± 10.3% to 56.0 ± 6.6%; p = 0.01) and between 24 and 48 hpi (from 56.0 ± 6.6% to 79.2 ± 8.7%; p = 0.0006) (Fig 2D). The maximum percent of infected cell bodies was observed at 48 hpi with no significant increase at 72 hpi (84.0 ± 7.0%) or 168 hpi (86.7 ± 8.8%). To verify the P-mCherry fusion in infected cells, we collected cell bodies at 72 h post axonal infection and used western blotting with anti-mCherry antibody. We observed a major band at the expected size for the P-mCherry fusion protein (~61 kDa) but no band for the unfused mCherry protein (~27 kDa) (Fig 2E). Thus, RABV enters SCG axons and moves retrograde to infect approximately half of the connected cell bodies within 24 h. The infected cell bodies express the intact P-mCherry fusion protein.

### Retrograde RABV infection is unaffected by axonal interferon treatment

**Fig 3:**
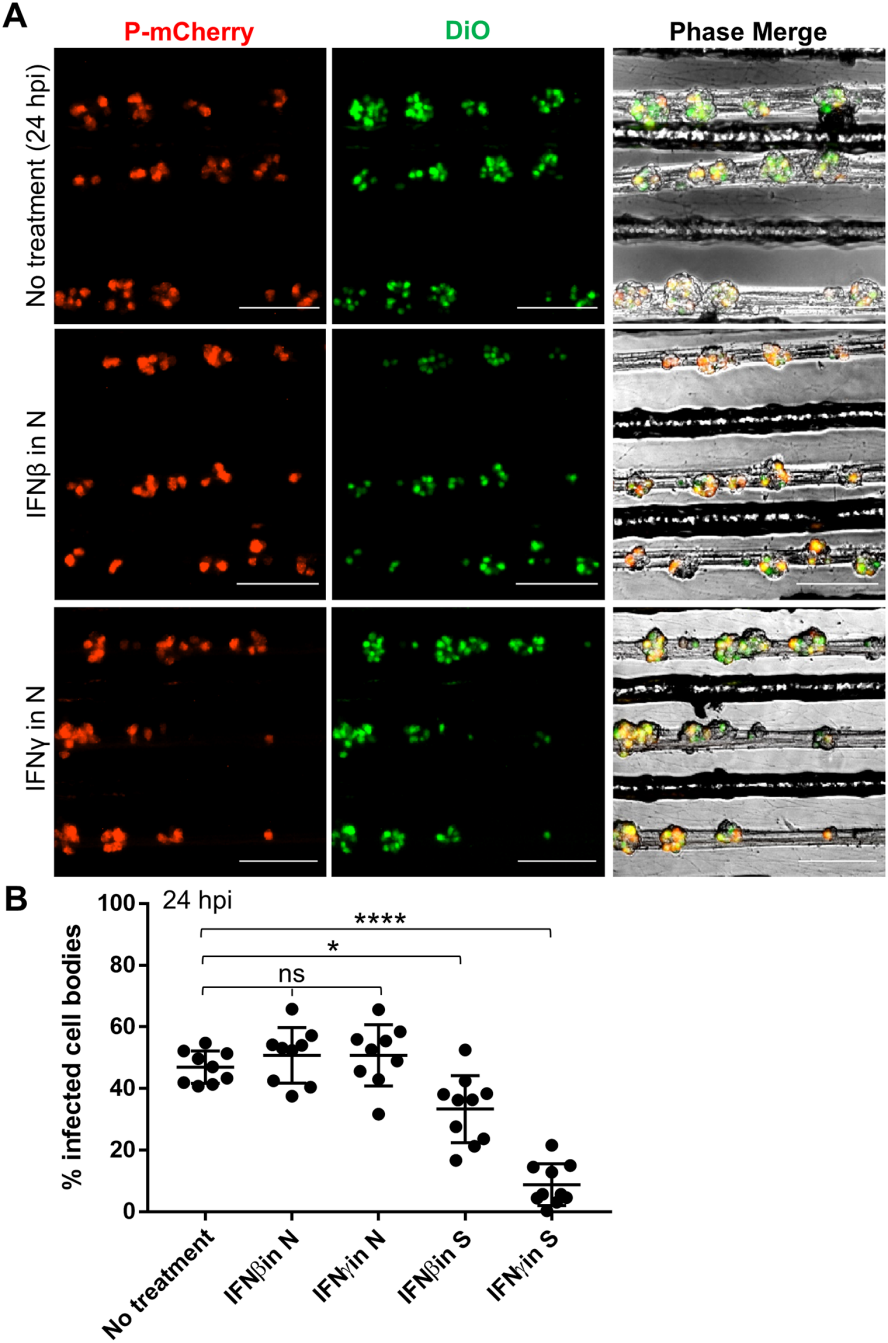
Retrograde RABV infection is unaffected by axonal interferon treatment. **(A)** Cell bodies in S at 24 h post RABV P-mCherry infection in N. IFNβ or IFNγ was added to N for 24 h prior to axonal infection (scale bars = 250 μm). **(B)** Quantification of % infected cell bodies at 24 hpi (+/-) IFNβ or IFNγ in N or S. Black dots represent individual tri-chambers (from three independent replicates). Horizontal lines and errors bars represent mean ± SD for each condition with *p = 0.0114, ****p < 0.0001 using one- way ANOVA (ns = not significant).

We have shown previously that pre-exposure of SCG axons to either type I (IFNα/β) or type II (IFNγ) interferon reduces the axonal transport of αHV particles but not lysotracker-positive vesicles [8]. Because RABV and αHV particles use distinct axon entry/transport mechanisms (in endosomes (RABV) vs. vesicle-independent (αHV)), we determined if exposing axon termini to these pro-inflammatory cytokines would also restrict retrograde infection by RABV. Axons in the N compartment were pretreated with IFNβ or IFNγ for 24 h prior to infection with RABV P-mCherry. At 5 hpi, DiO was added to the axon compartment to label the connected cell bodies (Fig 2A). At 24 h post axonal infection, there was no significant difference in P-mCherry expression in the cell bodies regardless of whether axons were pretreated with IFNβ, IFNγ or untreated (Fig 3A). P-mCherry was expressed in 47.0 ± 5.3% of the connected cell bodies in the control condition and in 50.8 ± 9.0% and 50.8 ± 9.9% of the connected cell bodies in the IFNβ and IFNγ pretreated conditions, respectively (Fig 3B).

To verify that IFNβ and IFNγ were active, we added IFNβ or IFNγ directly on the cell bodies in the S compartment 24 h prior to axonal infection. Consistent with previous reports of interferon inhibiting RABV replication [30,31], we observed a statistically significant decrease in P-mCherry expression in cell bodies that were directly pretreated with either IFNβ or IFNγ. The percentage of infected cell bodies decreased from 47.0 ± 5.3% (untreated) to 33.4 ± 10.9% (p = 0.0114) and 8.83 ± 6.8% (p < 0.0001) when cell bodies were exposed to IFNβ or IFNγ, respectively (Fig 3B). IFNβ pretreatment of cell bodies did not completely abolish RABV infection, suggesting that RABV P-mCherry maintains its IFN antagonist activity [32,33].

As an additional control for IFN activity, we measured phosphorylation of STAT1 (signal transducer and activator of transcription 1), a downstream effector of the IFN response. IFNβ or IFNγ was added to the N compartment, and after a 24 h treatment, the N and S compartments were lysed separately and analyzed by western blot. Consistent with previously published results [8], axonal IFNβ treatment induced phosphorylation of STAT1 only in axons, whereas axonal IFNγ treatment induced accumulation of phosphorylated STAT1 in the distant cell bodies (S2 Fig).

### Axonal interferon treatment does not alter retrograde axonal transport of RABV particles

**Fig 4:**
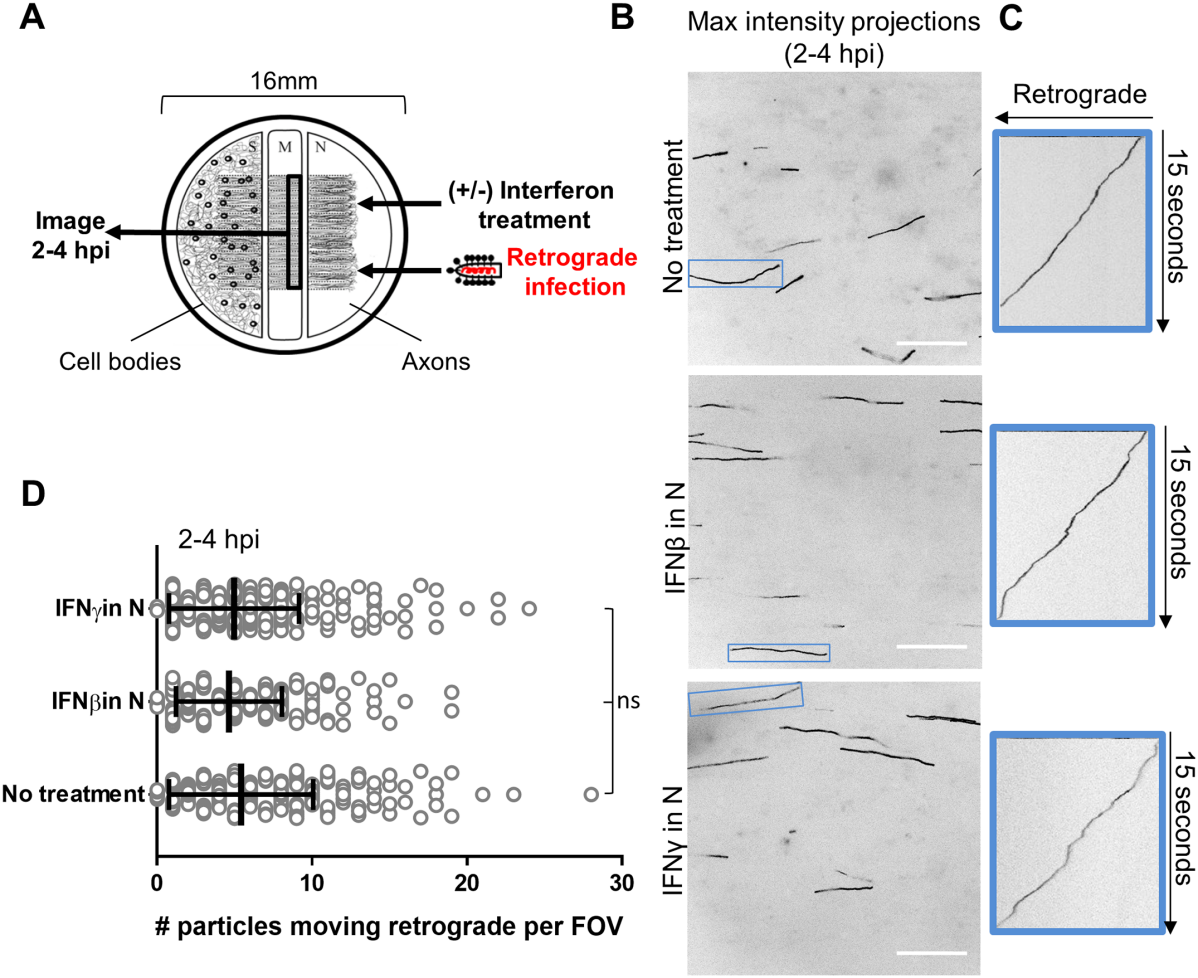
Axonal interferon treatment does not alter retrograde axonal transport of RABV particles. **(A)** Live particle tracking setup (see also S3 Fig). IFNβ or IFNγ was added to N for 24 h prior to infection in N. **(B)** Motile RABV particle tracks (black lines) visualized by maximum intensity projections from each field of view (FOV) along the M compartment barrier at 2–4 hpi in N (scale bars = 20 μm). One FOV is shown per condition. Blue boxes highlight tracks of particles that moved retrograde for the entire duration of the 15 sec movie (163 frames). **(C)** For tracks in blue boxes, kymographs show the displacement of each particle over time. Diagonal lines indicate particles moving retrograde. **(D)** Quantification of the number of RABV particles moving retrograde per FOV (+/-) IFNβ or IFNγ pretreatment in N. Each open circle represents an individual FOV. Vertical lines and error bars represent the mean ± SD for each condition (ns = not significant using one-way ANOVA). Total FOVs counted were 308 (no treatment), 297 (IFNβ), and 309 (IFNγ) across 3 independent replicate chambers per condition.

To confirm that axonal IFN exposure has no effect on retrograde RABV infection, we used time-lapse video microscopy with high temporal resolution (> 10 frames/sec) to track the number of RABV particles transported retrograde into the M compartment in the presence or absence of IFNβ or IFNγ between 2–4 h post-axonal infection (Fig 4A). We first confirmed the identity of the transported P-mCherry particles in M compartment axons by anti-RABV N staining (S3 Fig). We then visualized and counted the tracks of moving RABV particles using maximum intensity projections of each field of view (FOV) along the M compartment barrier (Fig 4B). We constructed kymographs to visualize the displacement of individual RABV particles over time during 15 second movies (Fig 4C). In all treatment conditions, particles moved with similar kinetics and relatively constant velocities. There was no significant difference in the average number of particles moving retrograde per FOV across the untreated (5.4 ± 4.7 particles), IFNβ pretreated (4.7 ± 3.4 particles) and IFNγ pretreated (5.0 ± 4.2 particles) axons (Fig 4D). Unlike the effect on αHV axonal infection, exposure of axons to IFNβ or IFNγ does not reduce retrograde RABV infection in the connected cell bodies nor does it alter axonal transport dynamics of RABV particles.

### Axonal emetine treatment blocks retrograde RABV infection

**Fig 5:**
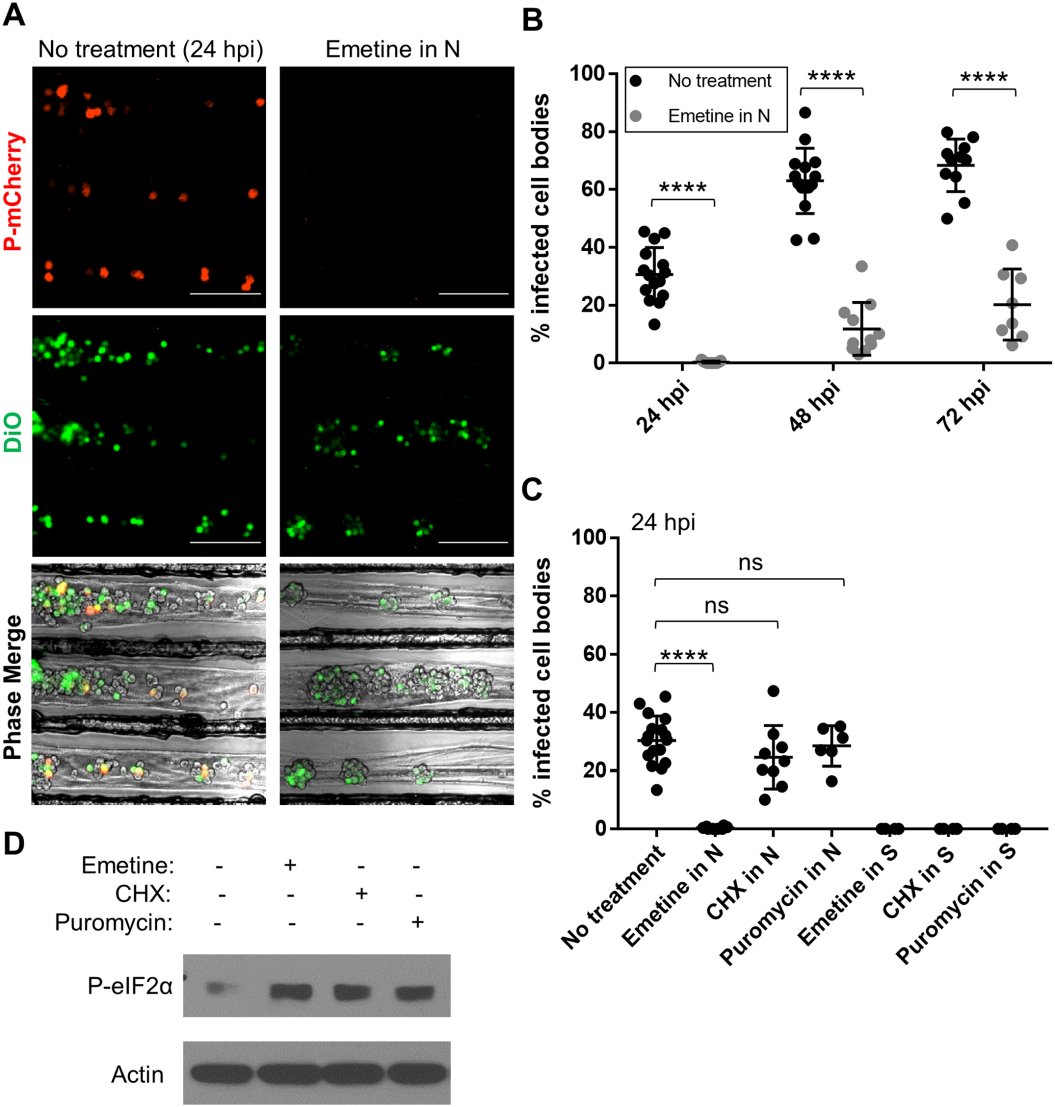
Axonal emetine treatment blocks retrograde RABV infection by a mechanism that does not depend on protein synthesis inhibition. **(A)** Cell bodies in the S compartment at 24 h post RABV P-mCherry infection in N. Emetine (100 μM) was added to N, 1 h prior to axonal infection and washed out at 5 hpi (scale bar = 250 μm). **(B)** Quantification of % infected cell bodies at 24, 48, and 72 hpi (+/-) 100 μM emetine in N. **(C)** Quantification of % infected cell bodies at 24 hpi (+/-) 100 μM emetine, 100 μg/ml CHX, or 10 μg/ml puromycin added to N or S, 1 h prior to infection in N. Inhibitors were washed out at 5 hpi. Black dots in (B) and (C) represent individual tri-chambers (from four (B) and three (C) independent replicates). Horizontal lines and errors bars represent mean ± SD for each condition with ****p < 0.0001 using two-way (B) or one-way (C) ANOVA (ns = not significant). **(D)** Levels of phosphorylated eIF2α (P-eIF2α) in dissociated SCG (+/-) 100 μM emetine, 100 μg/ml CHX or 10 μg/ml puromycin (6 h post treatment).

We previously reported that retrograde αHV infection requires local protein synthesis in axons, and αHV transport is reduced by treatment of isolated axons with protein synthesis inhibitors [7]. To determine if inhibition of axonal protein synthesis blocks retrograde RABV infection, N compartment axons were pretreated with the protein synthesis inhibitor, emetine, 1 h prior to RABV infection (see Fig 2A). Emetine-containing media was removed 5 hpi. We observed a remarkable decrease in the percentage of P-mCherry-positive cell bodies in the emetine-treated samples when compared to untreated samples, and this effect was dose-dependent (Fig 5A and 5B; see also S4A Fig). At 24 hpi, the percentage of infected cell bodies was 29.4 ± 8.5% in the untreated condition versus 14.0 ± 6.7%, 4.5 ± 2.0%, and 0.01 ± 0.02% in the 10 μM, 50 μM, or 100 μM emetine-treatment conditions, respectively (S4A Fig).

In axons treated with 100 μM emetine, retrograde infection was essentially blocked at 24 hpi (Fig 5B). The percentage of infected cell bodies remained significantly decreased for 72 hpi when compared to the control. However, a smaller proportion of cell bodies did become infected at later time points. In the emetine treated condition, P-mCherry was expressed in 12 ± 9% of the connected cell bodies at 48 hpi (vs. 63.0 ± 11.3% in 48 hpi untreated), and 20 ± 12% of the connected cell bodies at 72 hpi (vs. 68.4 ± 9.1% in 72 hpi untreated).

We verified that the observed decrease in retrograde infection was due to local effects of emetine on isolated axons and not due to diffusion of the drug to the connected cell bodies. N compartment axons were pretreated with emetine, but in this case, S compartment cell bodies were directly infected with RABV at 1 h post axonal emetine treatment. At 24 hpi, there was no significant difference in the percentage of infected cell bodies between the control and emetine-treated conditions (80.21 ± 12.5% and 83.14 ± 6.9%, respectively) (S4B Fig). Thus, when emetine is added to the N compartment, it acts locally in axons to block retrograde RABV infection in a dose-dependent manner.

To rule out the possibility that axonal emetine treatment has toxic effects, we first examined the morphology of axons and cell bodies at 24 h after axons had been treated with emetine for 6 h. Regardless of whether axons were treated with 100 μM emetine or untreated, the axons and cell bodies were intact and healthy with no apparent toxicity (e.g. no varicosity formation or blebbing) (S5A Fig). We then used SYTOX, a nucleic acid stain that is impermeant to live cells, to further analyze the percentage of live versus dead cell bodies after axons were treated with 100 μM emetine for 6 h. After a 6 h treatment, we counted a similar percentage of dead cell bodies in the emetine-treated condition (3.9% dead cells) versus the untreated (3.2% dead cells) (S5B and S5C Fig). We also imaged the cells at 24 h after a 6 h treatment with emetine in the N compartment (i.e. emetine was washed out at 6 h) and found no increase in cell death after 24 h (3.5% dead cells). By contrast, when emetine was added directly to the cell bodies in the S compartment, we observed a substantial increase in the percentage of dead cells after a 6 h treatment (14.4% dead cells) and a longer 24 h treatment (46.5% dead cells). Therefore, exposing isolated axons to 100 μM emetine for 6 h causes no apparent toxicity.

### Emetine blocks retrograde RABV infection by a mechanism that does not depend on protein synthesis inhibition

Because emetine is a translation elongation inhibitor, we hypothesized that the early steps of RABV entry and/or retrograde transport in axons require local, axonal protein synthesis to proceed efficiently. To test this hypothesis, we exposed axons to two other well-known protein synthesis inhibitors and measured the extent of retrograde RABV infection. Axons in the N compartment were treated with either the translation elongation inhibitor, cycloheximide (CHX) or the polypeptide chain terminator, puromycin, 1 h before axonal infection. Unexpectedly, neither of these inhibitors blocked retrograde RABV infection. In the CHX- and puromycin-treated conditions, 24.6 ± 10.9% and 28.5 ± 6.9% of the connected cell bodies became infected compared to 30.4 ± 8.4% in the control at 24 hpi (Fig 5C). By contrast, emetine treatment reduced the percentage of infected cell bodies to 0.4 ± 0.5%.

To confirm that the protein synthesis inhibitors were active at the concentrations tested, we added emetine, CHX or puromycin to cell bodies in the S compartment and infected the N compartment with RABV 1 h post inhibitor treatment. Retrograde RABV infection was completely abolished when each of these drugs was added directly to the S compartment, suggesting that CHX and puromycin actively block viral replication in cell bodies despite having no local effect in axons (Fig 5C).

We further confirmed translation inhibition by assessing the phosphorylation state of eIF2α (eukaryotic initiation factor 2 alpha) in dissociated SCG treated with emetine, CHX, or puromycin for 6 h. The level of phosphorylated eIF2α increased in each drug-treated condition as compared to the non-treated condition (Fig 5D). Because retrograde RABV infection is blocked exclusively by emetine but not by other protein synthesis inhibitors, we conclude that emetine inhibits axonal infection by an alternative mechanism that is independent of cytosolic protein synthesis inhibition.

### Emetine limits axonal transport of RABV particles but does not restrict transport of Rab5- or Rab7-positive vesicles

**Fig 6:**
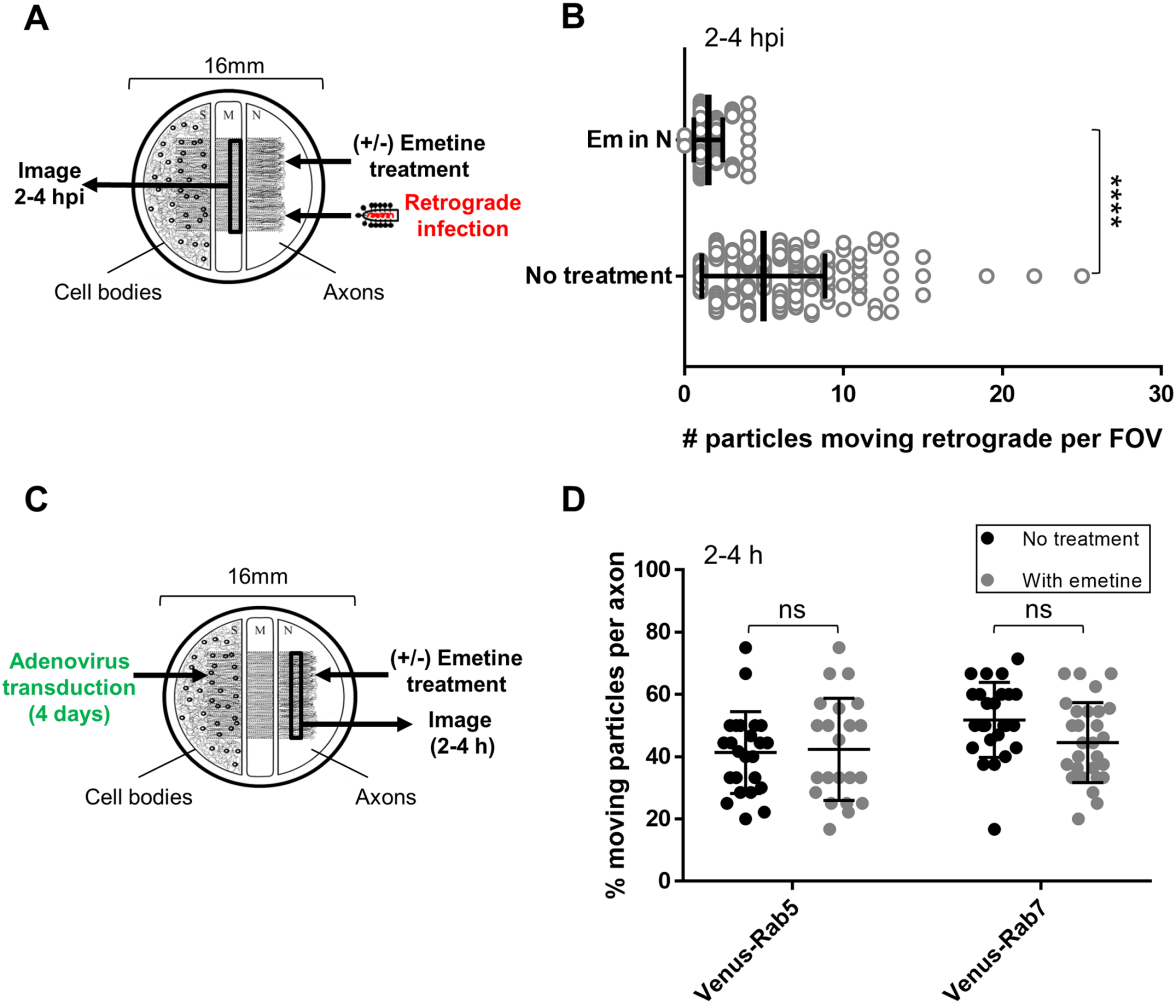
Emetine restricts axonal transport of RABV particles but does not limit transport of Rab5- or Rab7-positive vesicles. **(A)** 100 μM Emetine was added to N, 1 h prior to RABV P-mCherry infection in N. **(B)** Quantification of the number of RABV particles moving retrograde per FOV (+/-) emetine pretreatment in N. Open circles represent individual FOVs along the M compartment barrier. Vertical lines and error bars represent the mean ± SD for each condition with ****p < 0.0001 using unpaired t-test. Total FOVs counted were 224 (no treatment) and 166 (emetine) across 3 independent replicate chambers per condition. **(C)** S compartment cell bodies were transduced with adenoviruses expressing either Venus-Rab5 or Venus-Rab7 for 4 days. **(D)** Quantification of % moving Rab5 or Rab7 particles per axon (+/-) 100 μM emetine. Each dot represents one axon in the N compartment. A minimum of 22 axons were imaged per condition. Horizontal lines and error bars represent the mean ± SD for each condition (ns = not significant using two-way ANOVA).

We used video microscopy to determine if emetine blocks retrograde infection by limiting the number of RABV particles transported toward the cell bodies. We infected N compartment axons with RABV P-mCherry in the presence or absence of emetine and imaged along the M compartment barrier between 2 and 4 hpi to count the number of particles moving toward the cell bodies (Fig 6A). In the presence of emetine, we observed a significant decrease in the number of particles moving retrograde per FOV in the M compartment. We counted 5.0 ± 3.9 moving particles/FOV in the untreated axons versus 1.5 ± 0.9 in the emetine pretreated axons (p < 0.0001) (Fig 6B).

To determine whether emetine inhibits global axonal transport, we tested emetine’s effect on the transport of vesicles containing Rab5 (Ras-related protein 5) or Rab7, which localize to early and late endosomes, respectively [34]. Cell bodies in the S compartment were transduced with adenoviruses expressing either Venus-Rab5 or Venus-Rab7 fusion protein (Fig 6C). At 4 days post transduction, emetine was added to the N compartment (in the absence of RABV infection), and axons in the N compartment were imaged at 2–4 h post treatment. We calculated the percentage of Rab5 or Rab7 particles moving per axon and found no significant difference between the emetine pretreated and untreated axons (Fig 6D). The percent of Rab5-positive vesicles moving per axon was 41 ± 13% in the control versus 42 ± 16% in the emetine treated axons. For Rab7, the percent of vesicles moving per axon was 52 ± 12% (control) versus 45 ± 13% (emetine). Thus, emetine specifically affects RABV and does not reduce transport of cellular vesicles.

### Emetine acts after RABV entry to reduce the velocity and transport distance of virus particles moving retrograde in axons

We next determined whether emetine affects axon entry of RABV particles. If emetine’s primary effect was on RABV entry, we expected that retrograde infection would be unaltered if emetine was added to the N compartment after viral entry occurred. To define the window of time for the majority of RABV entry events to occur, we measured how long virus inoculum must be in contact with axons to establish an efficient retrograde infection in the cell bodies within 24 h. Axons in the N compartment were incubated with RABV P-mCherry inoculum for 1 min, 10 min, 30 min, 60 min, 120 min, or 300 min (S6A Fig). The percentage of infected cell bodies was 0% after 1 min, 3.1 ± 1.1% after 10 min, 14.0 ± 5.2% after 30 min, 25.7 ± 4.4% after 60 min, 25.1 ± 1.1% after 120 min, and 28.6 ± 4.7% after 300 min (S6B Fig). Incubations longer than 60 min did not significantly increase the percentage of infected cells. Therefore, 60 min provides a suitable time window for the majority of axon entry events to occur.

**Fig 7:**
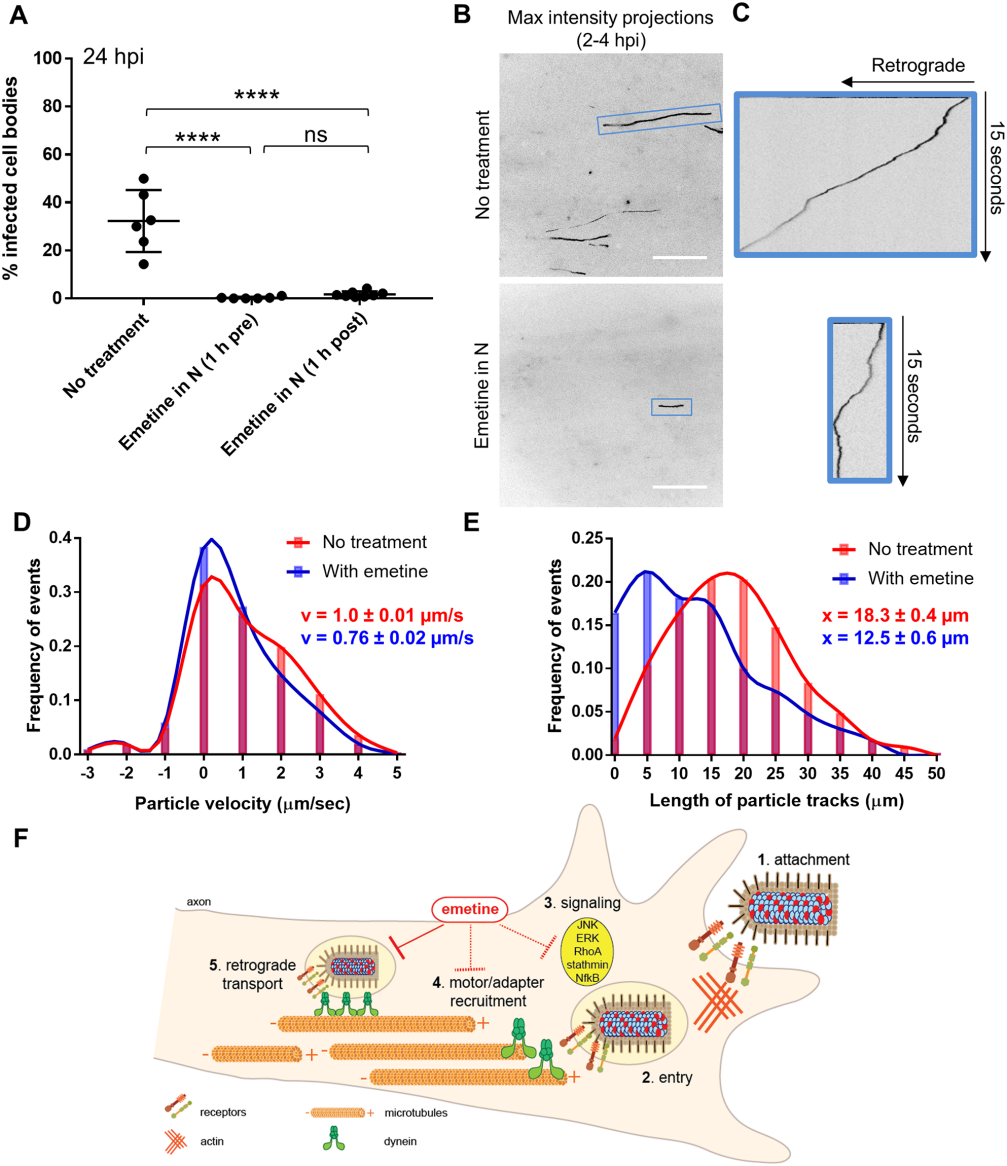
Emetine acts after RABV entry to reduce the velocity and transport distance of virus particles moving retrograde in axons. **(A)** Quantification of % infected cell bodies at 24 hpi (+/-) 100 μM emetine added to N either 1 h pre or 1 h post infection. Black dots represent individual tri-chambers (from two independent replicates). Horizontal lines and errors bars represent mean ± SD for each condition with ****p < 0.0001 using one-way ANOVA (ns = not significant). **(B)** Motile RABV particle tracks (black lines) visualized by maximum intensity projections from each FOV along the M compartment barrier at 2–4 hpi in N (scale bars = 20 μm). **(C)** For tracks in blue boxes, kymographs show the displacement of particles over time during the 15 sec movie (163 frames). The slope indicates the velocity of particle movement, where a vertical line has a slope of zero and indicates particle stalling. **(D)** Distribution of RABV particle velocity (μm/sec) across a population of retrograde moving RABV particles in the untreated (red; n = 10300 constant velocity segments from 1116 events) or emetine-pretreated (blue; n = 3363 constant velocity segments from 338 events) condition. Events were pooled from three independent replicates. Y axis indicates the frequency for each velocity on the x-axis. Positive and negative values indicate retrograde and anterograde directed motility, respectively. v = the mean velocity ± standard error of the mean (SEM) **(E)** Distribution of RABV particle track length for particles moving retrograde in the untreated (red; n = 637 particles) or emetine-pretreated (blue; n = 231 particles) conditions. Y axis indicates the frequency for each track length on the x-axis. x = the mean particle track length (± SEM). Lines on the histograms in (D) and (E) are cubic spline curves. **(F)** Our suggested model summarizing the effect of emetine on post-entry retrograde transport of RABV virions. RABV particles first attach to the cell surface receptors (step 1) to initiate entry through endocytosis (step 2). Step 1 and 2 induce signaling pathways including JNK, ERK, RhoA, stathmin and NfkB in axons (step 3). RABV-carrying endosomes must recruit dynein motors and adapters (step 4) to facilitate efficient retrograde transport on microtubules (step 5). We propose that emetine does not block step 1 or 2, but it interferes with step 5 by possibly inhibiting step 3 and/or 4.

We found that retrograde RABV infection in the cell bodies was almost completely abolished regardless of whether emetine was added to axons prior to or after infection. When emetine was added to axons 1 h after infection, we still observed a significant decrease in the percentage of infected cell bodies from 32.3 ± 12.9% (untreated) to 2 ± 1% (p < 0.0001) (Fig 7A). Findings suggest that the primary antiviral effect of emetine is not on RABV entry into axons.

We next determined if emetine alters the dynamics of RABV transport by modifying the velocity or transport distance of moving particles. We visualized tracks of RABV particles moving in the retrograde direction by maximum intensity projections of each FOV in the M compartment at 2–4 h after N compartment infection (Fig 7B). From individual tracks, we constructed kymographs to characterize the motion of particles during 15 second movies (163 frames) (Fig 7C). The velocity of particle movement is represented by the slope of the track in the kymograph. In the emetine-treated condition, kymograph analysis indicated periods of decreased slope (closer to zero) with more frequent stalling as compared to the control track (Fig 7C).

To understand how emetine affects the velocity of moving RABV particles across the entire population, we parsed individual particle tracks from M compartment movies into segments of constant velocity and calculated the average instantaneous velocity for each segment. RABV particles moved slower in the emetine pretreated condition (0.76 ± 0.02 μm/s) versus the control condition (1.0 ± 0.01 μm/s) (Fig 7D). To determine how far RABV particles traveled in the retrograde direction in the absence and presence of emetine, we measured the length of each particle track. RABV particles traveled shorter distances in the emetine condition (12.5 ± 0.6 μm) versus the control (18.3 ± 0.4 μm) (Fig 7E). Taken together, emetine treatment significantly reduces the number of RABV particles transported toward the cell bodies. For the minority of particles that still move in the retrograde direction in the presence of emetine, they move with altered transport dynamics, including slower velocities and shorter distances.

## Discussion

Nervous system infection is usually a dead end for many viruses; the host dies and viral transmission is terminated. Yet viruses of the *Rhabdoviridae* family and the alpha *herpesvirinae* subfamily, have evolved strategies that exploit neuronal biology to efficiently infect the nervous system and still preserve host-to-host transmission. Recent work has revealed that RABV hijacks the neurotrophin signaling pathway, and αHVs repurpose the axon damage response to invade peripheral nervous system axons [7,16]. Both strategies achieve a common goal: the efficient dynein-mediated retrograde transport of viral particles from axon terminus to cell body where viral replication ensues. Due to the extreme spatial separation of axons from cell bodies, the axon is a neuronal sub-compartment that senses external stimuli and mounts independent local responses. An outstanding question is do such axonal responses regulate or limit axonal infection by two distinct neuroinvasive viruses?

Previous studies have investigated RABV axonal transport using attenuated vaccine strains (SAD B19) or RABV G-pseudotyped vesicular stomatitis virus and lentivirus [11,12,16,18,35]. In this study, we extend the current understanding of RABV axon transport dynamics by use of a neurovirulent CVS-N2c-derived RABV P-mCherry-expressing recombinant. We characterize RABV infection in SCG sympathetic neurons grown in compartmented neuronal cultures, in which the physical separation of cell bodies enables selective infection or treatment of isolated axons. Although somatic motor neurons are predominantly infected by RABV *in vivo* [36,37], the autonomic motor neurons of the SCG are a homogenous population that provides a good model of the early steps of neuroninvasion due to highly reproducible and quantifiable axonal infections. To date, sensory dorsal root ganglia (DRG) or ventral spinal cord neurons have been used for RABV axonal transport studies. However, unlike SCG, spinal cord neurons exhibit limited axonal projections in compartmented cultures [12], and DRG are heterogenous with subpopulations of unmyelinated neurons that display resistance to RABV infection [38]. We established an *ex vivo* model of RABV infection in compartmented SCG neurons to facilitate high resolution imaging of RABV axon transport as well as concurrent biochemical analyses of RABV-infected axons.

Using this system, we showed that SCG axons do not regulate RABV and αHV invasion in the same way. We previously reported that treatment of axons with either type I IFN (IFNβ) or type II IFN (IFNγ) was sufficient to limit retrograde αHV infection by reducing the percentage of moving αHV particles by at least 50%. Exposure of axons to IFNβ induced a strictly local axonal response where STAT1 was phosphorylated and retained in axons. Whereas, exposure of axons to IFNγ induced a long distance signaling response where phosphorylated STAT1 was present in the connected cell bodies but not the axons. Importantly, axonal IFN exposure had no effect on the transport of cellular lysosomes, suggesting that vesicular transport was unaffected [8].

As expected, proinflammatory cytokine pretreatment of neuronal cell bodies moderately suppressed RABV infection in this compartmented system. However, exposure of isolated axons to IFNβ or IFNγ, had no effect on RABV neuroinvasion. Why does axonal IFN exposure restrict αHV transport but have no effect on RABV? The most apparent explanation is that RABV is shielded from the axonal inflammatory response due to endosome-mediated transport. Previous studies in hippocampal neurons [39], motor neurons [35], and dorsal root ganglia (DRG) neurons [12,16] showed that clathrin-mediated endocytosis is the primary entry mechanism for RABV particles. In contrast, the primary entry mechanism for αHV is membrane fusion followed by release of non-enveloped viral capsids and tegument proteins into the axoplasm [40]. αHV particles interact directly with dynein and are not transported inside vesicles [41]. As a result, αHV are likely to be more overtly susceptible to IFN-induced axonal responses.

The contrasting effects of axonal IFN exposure on RABV and αHV likely reflect differences in how these viruses evolved with their hosts. Unlike RABV which is 100% lethal after infection of most mammals, αHV establish a lifelong latent infection in the PNS ganglia of the host. Given the importance of latency establishment for αHV persistence, it is possible that αHV evolved to exploit the innate immune response to bias the infection mode toward latency. Interestingly, axonal IFN exposure reduces the number of αHV particles transported toward the cell bodies, which leads to silencing of herpesviral genomes in the neuronal nuclei [42,43]. Such exposure of isolated axons to IFN is biologically relevant for αHV because the initial replication in mucosal epithelia triggers the production and release of proinflammatory cytokines that bathe the innervating axon termini. Perhaps RABV encounters a different inflammatory milieu than αHV in its natural host, giving rise to distinct evolutionary pressures that affect how these viruses respond to innate defenses.

Previous studies have shown that axons respond not only to inflammatory cytokine exposure, but also to damage, axon guidance cues, and αHV infection by local translation of specific pools of mRNAs [5,7,44]. However, axonal RABV infection did not induce detectable new protein synthesis, and there was no effect on RABV infection when we treated axons with cycloheximide or puromycin. By contrast, we found that emetine dramatically blocked retrograde RABV infection in a dose-dependent manner. We first observed that axonal emetine pretreatment strongly limited infection in the connected cell bodies, reducing the percentage of infected cells from 30% to less than 0.5%. Importantly, retrograde infection was still blocked when emetine was added to axons 1 h after infection, suggesting that emetine functions after RABV particles enter axons. Furthermore, axonal emetine pretreatment caused a 70% decrease in the number of particles moving in the retrograde direction into the middle compartment. The few particles that still moved in the presence of emetine, moved an average of 25% slower and traveled approximately 5.5 μm less than particles in untreated samples. The effect of emetine is not due to a global inhibition of axon transport because in the absence of RABV infection, emetine does not limit the proportion of moving Rab5- or Rab7-positive vesicles in axons. Emetine was the only protein synthesis inhibitor that blocked infection, suggesting that protein synthesis inhibition is not the primary mechanism by which emetine inhibits retrograde RABV infection.

A number of groups have recently reported new antiviral roles for emetine. Emetine was shown to inhibit viral nucleic acid synthesis in cultured cells infected with the RNA viruses, PPRV (peste des petits ruminants virus) and NDV (Newcastle disease virus), or the DNA viruses, BPXV (buffalo poxvirus) or BHV-1 (bovine herpes virus). Entry of NDV-1 and BHV-1 was also inhibited by emetine [45]. Furthermore, emetine inhibited human cytomegalovirus (HCMV) replication by disrupting the HCMV-induced interaction between p53 and the E3-ubiquitin ligase, MDM2. Importantly, this effect was not attributed to protein synthesis inhibition because translation was not inhibited at the concentrations used [46].

Recent reports, particularly in the cancer literature, have introduced emetine as an important modulator of cellular signaling pathways. In a screen of ~2800 clinically approved drugs, emetine was found to block NF-кB (nuclear factor-kappa B) activation via inhibition of IкBα phosphorylation in human cervical cancer cell lines [47]. A separate study showed that emetine promotes splicing of Bcl-x to a pro-apoptotic variant in several tumor cell lines, and this action is dependent on the emetine-induced activation of protein phosphatase 1 (PP1). Notably, the authors concluded that this effect of emetine on splicing was not likely to be dependent on protein synthesis inhibition because several other protein synthesis inhibitors did not have analogous effects [48]. In human non-small-cell lung cancer cells (NSCLC), emetine treatment inhibited NSCLC migration and invasion via selective down-regulation of matrix metalloproteases that degrade the extracellular matrix. The mechanism underlying this effect of emetine was an increased phosphorylation of p38 MAPK (mitogen-activated protein kinase) and decreased phosphorylation of ERK (extracellular signaling regulated kinase) [49]. Interestingly, ERK1/2-mediated phosphorylation of the dynein intermediate chain was reported to enhance dynein recruitment to signaling endosomes and to promote retrograde axonal transport of these organelles [50].

We propose that emetine alters local signaling events in axons that are triggered by RABV infection and required for efficient retrograde transport of RABV (Fig 7F). Gluska et al. 2014 recently reported that retrograde transport of RABV was faster than transport of nerve growth factor (NGF), even though RABV exploited the endogenous NGF entry/transport route mediated by p75 neurotrophin receptor (p75NTR). The authors suggest that the increased RABV transport velocity is due either to recruitment of extra motors via receptor clustering at the membrane or to alteration of intra-axonal signaling pathways (e.g. JNK, RhoA, Stathmin, NfкB) downstream of receptor binding. We observed that emetine had no effect on virion entry but decreased the transport velocity of retrograde moving RABV particles. Perhaps an emetine-induced modulation of axonal signaling events prevents effective cargo-motor loading or motor recruitment to the abnormally large RABV-containing endosomes (Fig 7F). RABV-induced axonal signaling events and the effects of emetine on axonal signaling are currently under investigation.

In summary, we show that axons have unique responses to two different viral infections. Unlike what was previously reported for αHV, axonal protein synthesis is not required for RABV neuroinvasion, and RABV transport is not hindered by exposure of axons to interferon. Although both RABV and αHV repurpose the existing axonal transport machinery to establish efficient nervous system infection, the regulation of this repurposing is distinct between these two viruses. Furthermore, we show that exposure of axons to emetine efficiently blocks RABV infection in the distant cell bodies. Although we have not fully elucidated the mechanism, to our knowledge, this is the first study to show antiviral effects of emetine against RABV infection. These findings extend our current understanding of how viral neuroinvasion by two distinct viruses is regulated and point toward a novel role for emetine as an inhibitory modulator of RABV axonal transport.

## Materials and methods

### Animals

Timed-pregnancy Sprague-Dawley rats (*Rattus norvegicus*) were obtained from Hilltop Labs Inc. (Scottsdale, PA). E17 rat embryos were harvested for isolation of superior cervical ganglia (SCG).

### Primary superior cervical ganglia culture

SCG neurons were cultured in tri-chambers as described previously [26]. Briefly, SCG were trypsin-digested, mechanically dissociated, and seeded in the S (soma) compartment of the tri-chamber. Neurons extend axons beneath two physical barriers through the M (methocel/middle) compartment and into the N (neurite) compartment. Primary neurons were maintained in Neurobasal media (ThermoFisher; 21103049) + 50X B-27 supplement (ThermoFisher; 17504044) + 100X Penicillin-Streptomycin-Glutamine (ThermoFisher; 10378016) + 80 ng/ml NGF (ThermoFisher; 13257019) with media change every 5–7 days as needed. Neurons were grown at 37°C, 5% CO_2_ for 3–4 weeks prior to infection or drug treatment.

### Cell lines and virus strains

Neuro2A (N2a) mouse neuroblastoma cells (American Type Culture Collection (ATCC)), N2c G expressing N2a cells (NG cells) (Matthias Schnell Lab), and pig kidney epithelial cells (PK15) (ATCC) were grown at 37°C, 5% CO_2_ and maintained in DMEM + 10% FBS + 1% Penicillin-Streptomycin (PS). RABV P-mCherry was recovered and propagated on N2c G-expressing N2a cells. PRV recombinants mCherry-UL35 (960) and UL25-mCherry were propagated on PK15 cells. When cell lines were infected with virus, FBS was reduced to 2% in the infection media. Virus strains used in this study include RABV N2cΔG P-mCherry (described below), PRV mCherry-UL35 (PRV 960) [51], and PRV UL25-mCherry [25].

### Antibodies and chemicals

Fluorescein isothiocyanate (FITC)-conjugated anti-Rabies monoclonal globulin recognizing the RABV N protein (FUJIREBIO Diagnostics, Inc. Malvern, PA; 800–092) was used at dilutions of 1:200 for immunofluorescence (IF) and 1:1000 for western blot (WB). Anti-Rabies virus glycoprotein antibody [1C5] (Abcam; ab82460) was used at 1:100 for IF. Rabbit polyclonal anti-RABV N (raised against purified ribonucleoprotein particles) was made by the Schnell Lab and used at 1:1000 for WB. Monoclonal anti-RABV P antibody was a gift from Danielle Blondel [52] and used at 1 μg/ml for WB. Polyclonal anti-RABV M (raised against a SLQTQRSEEDKDSSL peptide from the C-terminus of M) was made by the Schnell Lab and used at 1:2000 for WB. Anti-RABV G for WB was a mixture of 4 human monoclonal antibodies (4C12, 10H5, 8C5, 4H3) used at 1 μg/ml each. This was a gift from Scott Dessain at Lankenau Institute of Medical Research. DAPI (4’,6-diamidino-2-phenylindole) stain was used at 1:1000 for IF. Alexa Fluor 488-conjugated goat anti-mouse IgG (H+L) secondary antibody (Thermo Fisher; R37120) was used at 1:1000 dilution for IF. Anti-phosphorylated STAT1 (Cell Signaling Technology; 7649S) and anti-phosphorylated eIF2α (CST; 9721S) were used at 1:1000 for WB. Anti-β-Actin (Sigma; A1978) was used at 1:10,000 for WB. Horseradish peroxidase-conjugated anti-mouse and anti-rabbit secondary antibodies (KPL; 31430 and 65–6120) were used at 1:10,000 for WB.

Emetine dihydrochloride hydrate (Sigma Aldrich; 45160) was used at 100 μM (dissolved in water) unless otherwise specified. Cycloheximide (CHX) (Sigma Aldrich; C7698) was used at 100 μg/ml (dissolved in DMSO), and Puromycin (Invivogen; ant-pr) was used at 10 μg/ml (dissolved in DMSO). Emetine, CHX, or Puromycin were added 1 h prior to infection unless otherwise specified. For the untreated/ no treatment controls, the appropriate solvent (water or DMSO) was added 1 h prior to infection unless other timing is specified. Recombinant rat IFN-gamma protein (R&D Systems; 585-IF-100) and recombinant rat IFN-beta (PBL Assay Science; 13400–1) were used at 500 U/ml (retrograde infection) or 1000 U/ml (particle tracking). IFN treatment was initiated 24 h prior to infection. SYTOX Green Nucleic Acid Stain (Thermo Fisher; S7020) was used at 5 nM for 10 min to label dead cells. Fluorescent lipophilic dyes (DiO Green—Thermo Fisher; D275 and DiI Red—Thermo Fisher; D282) were used at 1:1000 dilution to label cell bodies with axonal connection to the N compartment.

### Cloning a full-length cDNA of the recombinant N2cΔG P-mCherry genome

The RABV recombinant was derived from the highly neuroinvasive and neurotropic CVS-N2c parental strain [21]. PCR primers were designed to amplify 3 DNA fragments: 1) RABV P, 2) mCherry, and 3) RABV M with the intergenic region (IGR) after M, including an XmaI restriction site. Primers were designed to facilitate insertion of DNA fragments into a pCAGGS mammalian expression vector [53] in the following order: P, mCherry, M-IGR. The following primers were used for PCR amplification:

N2c P: 5’-TTGGCAAAGAATTCGTCTCCGTACGACCATGAGCAAGATCTTTGTTA-3’ (fwd) and 5’-GAGCCGTCGCCGGAGCAGGATGTATAGCGATTC-3’ (rev);
mCherry: 5’-TACATCCTGCTCCGGCGACGGCTCTGGCATGGTGAGCAAGGGCGAG-3’ (fwd) and 5’-GAAAACTCGGTTACTTGTACAGCTCGTCCATG-3’ (rev);
N2c M-IGR: 5’-GTACAAGTAACCGAGTTTTCGAACTCAGTC-3’ (fwd) and 5’-GGGAAAAAGATCTCGTCTCGCTAGCCTTCCCGGGGTCTTTTGAG-3’ (rev)

PCR products contained 15–40 bp of overlapping ends to facilitate fragment assembly. The stop codon of P was mutated, and mCherry was fused to the C-terminus with a 5 amino acid spacer sequence (GGG GAC GGC TCT GGC) inserted between the end of P and the start of mCherry. PCR products were ligated into pCAGGS vectors using NEBuilder HiFi DNA Assembly Cloning Kit (New England Biolabs; E5520S) following the recommended protocol for assembly of DNA inserts into linear vectors. Briefly, the digested pCAGGS vector and the 3 PCR products were added to the HiFi DNA Assembly master mix in equimolar amounts (0.1 pmol/fragment) and incubated at 50°C for 1 h. The ligation product was transformed into NEB5α competent cells (NEB; C29871) following the manufacturer’s protocol, and minipreps were prepared from single colonies. P-mCherry-M-IGR inserts were verified by restriction digest and DNA sequencing. P-mCherry expression was confirmed by fluorescence microscopy after transient transfection of the mammalian expression vector into N2a cells.

Verified inserts were digested out of the pCAGGS subcloning vector by cutting at the SpeI site (C-terminus of P) and XmaI cloning site (flanking the 5’end of the glycoprotein gene), and inserts were gel purified. The P-mCherry-M-IGR insert was ligated into the SpeI/XmaI-digested N2c RABV genomic cDNA using T4 ligase (NEB; M0202S) overnight at room temperature (RT). Ligated genomes were transformed into NEB5α cells, and single colonies were picked for mini preps.

For RABVΔG, N2c RABV genomic cDNA was digested with SpeI and NheI (cloning site flanking the 3’-end of the glycoprotein gene) to remove the genomic region from the C-terminus of P to the end of the G gene. P-mCherry-M-IGR was digested out of the pCAGGS expression vector and re-ligated into the SpeI/NheI-digested N2c genomic cDNA. P-mCherry insertion and G deletion were confirmed by restriction digest, and maxi preps were produced from verified clones prior to virus recovery.

### Recovery of recombinant N2c P-mCherry RABV and production of virus stocks

The RABV recombinant was recovered on a complementing cell line expressing N2c G (NG cells). The cells were made by transfecting N2a cells with plasmid vectors pTET-off (Clontech; 631017) and pTRE2Hyg (Clontech; 631014) containing the N2c glycoprotein gene. Clones were selected with Geneticin and Hygromycin. Resistant clones were cultured in the absence of doxycycline and screened by immunofluorescence staining with polyclonal antibody against the RABV glycoprotein. Two clones that stained positive for RABV-G were selected (NG7 and NG13) for setting up virus recovery and for large scale virus growth. Briefly, NG13 cells in 6 well dishes were transfected with 1.7 μg of full length genomic cDNA plus 2.5 μg of a mix of pTIT-N, pTIT-P, pTIT-L, and pCAGGS-T7 combined in a 4:2:1:2 ratio, respectively. Plasmids (pTIT) expressing the SAD B19 N, P, and L proteins were provided by Matthias Schnell [23]. The DNA transfection reagent, X-tremeGENE 9 (Roche; 06365809001) was used following the recommended protocol. Following overnight incubation at 34°C, the transfection reagent was replaced with fresh infection media (DMEM +2% FBS + 1% PS). Transfected cells were passaged once in 6 well dishes and then transferred to T-175cm^2^ flasks for the remainder of the virus recovery period. The cells were kept at 34°C for the duration of virus recovery and passaged as needed with media change every 4 days.

Red fluorescent foci were visible as early as 8 days post transfection, and all cells expressed P-mCherry by approximately 21 days post transfection. Supernatants were collected for virus stocks at days 21, 24, and 28 post transfection. Supernatants were spun for 20 min at 3000 rpm (4°C) to pellet cell debris. Supernatants were filtered through 0.45 μm filters and concentrated using Amicon Ultra -15 Centrifugal Filter Units (100KDa NMWL) (Millipore; Z740210). Concentrator units were spun for 20 min at < 4000 x g. Concentrated virus stocks were kept at 4°C for immediate use or -80°C for long term storage. Each virus stock was titered in triplicate on N2a cells to determine the concentration (ffu/ml).

### Viral particle purification

Concentrated virus stocks were overlaid on a 20% sucrose cushion in either 13.2 ml (Beckman Coulter; 344059) or 38 ml (Beckman Coulter; 344058) thin wall, ultra-clear tubes. Tubes were spun in a Beckman Coulter ultracentrifuge at 25,000 rpm for 1.5 h using the either the SW 41 Ti (13.2 ml tubes) or SW 32 Ti (38 ml tubes) swinging bucket rotor (107,000 x g). The supernatant was aspirated, the remaining liquid decanted, and the tubes were spun upside down in 50 ml conical tubes to remove any residual liquid. Pellets were resuspended in PBS at 4°C overnight. 6% sucrose was added for cryopreservation.

### Construction of adenoviral vectors

Adenoviral vectors were constructed by Gateway recombination into pAd/CMV/V5-DEST vectors (Invitrogen; V49320). GFP was fused to the N-terminus of Rab5 or Rab7 as described previously [54].

### Western blotting

Dissociated SCG neurons or cell bodies from the S compartments were lysed in RIPA light buffer (50 mM Tris-HCL, pH 8.0; 150 mM NaCl; 5mM EDTA; 1% (v/v) NP40; 0.1% (w/v) SDS; 0.1% (v/v) Triton X-100) supplemented with 1 mM DTT and protease inhibitor cocktail (Sigma Aldrich; P1860). Lysates were incubated on ice for 20 min and centrifuged at 10,000 rpm (4°C) for 5 min. Supernatants were mixed with 5x laemmli buffer and heated for 10 min at 90°C. Axons from N compartments were directly lysed in 2x laemmli and heated. Proteins were separated by SDS-PAGE on 4–12% NuPAGE Bis/Tris gels. Proteins were transferred to nitrocellulose membranes (GE Healthcare; 45-004-002) using a Trans-Blot SD semi-dry transfer cell (Bio-Rad). Membranes were blocked in 5% non-fat dry milk powder in PBS-T (PBS-Tween, 0.1%) for 1 h at RT. Primary antibody was diluted in 1% milk powder in PBS-T. Membranes were incubated with primary antibody overnight at 4°C followed by PBS-T washes (3 x 10 min). Horseradish peroxidase-conjugated anti-mouse and anti-rabbit secondary antibodies (KPL) were diluted in 1% milk powder in PBS-T. Secondary antibody was added to the membrane for 1 h at RT followed by three PBS-T washes. Chemiluminescent substrate, Supersignal West Pico or West Dura (Pierce), was added to the membrane for 5 min. Protein bands were visualized by exposing the blot on HyBlot CL autoradiography film (Denville scientific; E3018). For detection of viral proteins in sucrose purified particles, proteins were separated by SDS-PAGE on 10% polyacrylamide Tris/Glycine gels. Primary anti-RABV antibodies were diluted in 10% BSA, and secondary antibodies were diluted in PBS-T + 5% milk powder.

### Immunofluorescence staining

For immunofluorescence staining of P-mCherry-positive RABV particles, supernatants were collected from G-expressing N2A cells (NG cells) at 7 d post RABV P-mCherry infection (MOI 0.1). Following filtration, concentration, and purification (20% sucrose cushion in PBS), supernatants were spotted on glass coverslips and left to adsorb to the glass for 15 min at 37°C. Particles were blocked without fixation for 1 h in DMEM + 10% FBS followed by 1 h staining with 1° antibody recognizing the RABV G protein and Alexa Fluor 488-conjugated 2° antibody (green). Particles were washed 3 times with DMEM for 5 min per wash prior to and after 2° antibody staining.

For immunofluorescence of dissociated SCG or M compartment axons, cells were fixed in 4% PFA at RT for 10 min, permeabilized in 0.25% Triton X-100 for 20 min at RT, and blocked in 3% BSA-PBS for 1 h. FITC-conjugated anti-RABV (N) antibody was diluted in 3% BSA-PBS and added to cells for 1 h followed by three 5 min washes in PBS + 0.05% Tween. DAPI was added to dissociated SCG in the third wash for 5 min.

### Retrograde infection in tri-chambers

Unless otherwise specified, N compartment axons were either untreated (appropriate solvent added to control), pretreated with IFNβ or IFNγ for 24 h, or pretreated with protein synthesis inhibitors for 1 h prior to axonal infection with RABV P-mCherry (see Fig 2A). N compartment axons were infected with 10^5^ ffu of RABV P-mCherry unless otherwise specified. At 5 hpi, green lipophilic dye (DiO) was added to the N compartment to label the connected cell bodies in the S compartment. Using live cell imaging, the entire S compartment was tile imaged at designated times post axonal infection for P-mCherry expression (red) and DiO staining (green). Total numbers of green and dual-colored cell bodies were counted manually using NIS Elements Advanced Research software (Nikon). The average number of DiO-positive cell bodies counted per chamber (n = 150 chambers) was 1981 cells. The % infected cell bodies refers to the percentage of DiO-positive cell bodies that were also P-mCherry-positive.

For protein synthesis inhibitor experiments, the inhibitors and virus inoculum were washed out and removed from the N compartment at 5 hpi, at which point the N compartment was replaced with fresh neurobasal media. For the IFN experiments, IFN and virus inoculum were not removed from the N compartment, and media was not replaced throughout the duration of the experiment.

### Single particle tracking in tri-chambers

For RABV particle tracking, N compartment axons were either untreated (control), pretreated with IFNβ or IFNγ for 24 h, or pretreated with 100 μM emetine 1 h prior to RABV P-mCherry infection in N (10^5^ ffu). Time lapse imaging was used to visualize and count the number of RABV particles that moved from N into M between 2–4 hpi. Each movie captured one field of view (FOV) for 15 seconds (sec) at > 10 frames/sec. For Rab5 and Rab7 particle tracking, S compartment cell bodies were transduced with adenoviruses expressing either Venus-Rab5 or Venus-Rab7. At 4 days post transduction, the N compartment axons were either untreated or treated with 100 μM emetine (in the absence of RABV infection). At 2–4 h post emetine treatment, Rab motility was recorded in N compartment axons. The percentage of moving Rab particles to total particles (moving + stationary) was calculated per axon for a minimum of 22 axons per condition.

### Image processing and analysis

Imaging was conducted on a Nikon Eclipse Ti inverted epifluorescence microscope using a Photometrics CoolSNAP ES2 CCD camera or an Andor iXon3 EMCCD camera (particle tracking). Images and movies were processed using NIS Elements Advanced Research software (Nikon) and Fiji Image J [55]. Comparative images were captured with the same exposure times. Brightness and contrast adjustments were applied to the entire image, and alternations were applied equally across comparative images. For particle intensity analysis, the Fiji threshold feature was used to identify red particles. The analyze particle feature was then used to measure the maximum gray value for each red particle identified in the threshold. Particle intensity (S1B Fig) represents the mean maximum gray value across all particles analyzed for each recombinant strain. To determine the percentage of particles containing G, measurements were redirected from the red threshold to the green (anti-G) channel. A maximum gray value was obtained in the green channel at the location of each particle in the red channel. A red particle was considered dual-colored, if the maximum green intensity was above the background signal. To analyze particle transport, Fiji Z project was used to construct maximum intensity projections of individual fields of view that contained moving viral particles. Each maximum intensity projection was combined into a stack with its original movie file using the Fiji concatenate feature. The transport distance of each particle was measured by manually tracing the course of each track in the concatenated stack using the Fiji segmented line feature. Particles were excluded from the transport distance analyses if they entered or exited the field of view during the 15 s movie. Kymographs were created from single particle tracks using the Fiji KymographBuilder plugin. For velocity measurements, particle tracks were separated into segments of constant velocity as described previously [56].

### Statistical analysis

All data were analyzed with Graphpad Prism 7.04 (GraphPad Software, La Jolla California USA, www.graphpad.com). Figure legends specify the statistical test applied for each analysis, as well as the measure of dispersion about the mean and the number of replicates used. Experiments were repeated three times unless otherwise specified. Standard deviation (SD) is reported as the error for all measurements in the results section except for particle velocities and particle track lengths. For those population level measurements, the standard error of the mean (SEM) is reported. Differences were considered statistically significant when p values were less than 0.05 (*p < 0.05, **p < 0.01, *** p < 0.001, ****p < 0.0001).

### Ethics statement

All animal work was conducted in accordance with the Institutional Animal Care and Use Committee (IACUC) of Princeton University Research Board. The IACUC approved all animal experiments (protocol # 1947). Animals were euthanized by carbon dioxide inhalation, as recommended by the American Veterinary Medical Association (AVMA) guidelines on euthanasia. All personnel adhered to applicable federal, state, local, and institutional laws and policies governing ethical animal research. This includes the Animal Welfare Act (AWA), the Public Health Service Policy on Humane Care and Use of Laboratory Animals, the Principles for the Utilization and Care of Vertebrate Animals Used in Testing, Research and Training, and the Health Research Extension Act of 1985.

## Supporting information

S1 Fig RABV P-mCherry particles contain an average of 116 copies of the P protein. **(A)** PRV UL25-mCherry, RABV P-mCherry, and PRV mCherry-UL35 particles from sucrose-purified supernatants (scale bars = 5 μm). **(B)** Quantification of particle intensity (arbitrary units (AU)) based on mCherry brightness across individual particles of PRV UL25-mCherry (n = 815), RABV P-mCherry (n = 825), and PRV mCherry-UL35 (n = 841). Error bars indicate mean maximum particle intensity + SD. **(C)** Table listing the approximate average copy number per particle of RABV P and PRV pUL35. Protein copy number was extrapolated from the known PRV pUL25 copy number (60 copies) by comparing the mCherry fluorescence emission intensities between virus strains.
(TIF)

S2 Fig Axonal IFNβ or IFNγ treatment induces STAT1 phosphorylation in axons or cell bodies, respectively. N compartment axons were treated with IFNβ or IFNγ for 24 h. S and N compartments were lysed separately, and proteins were separated by SDS-PAGE on 4–12% gradient gels. Phosphorylated STAT1 (P-STAT1) levels were determined in the S and N compartments by western blotting. Symbols indicate the presence (+) or absence (-) of IFN in the N compartment. Β-actin served as a loading control.
(TIF)

S3 Fig RABV P-mCherry particles that are transported retrograde to the M compartment co-stain with RABV nucleocapsid (N) protein. **(A)** Experimental setup for immunofluorescence (IF) staining of RABV particles in the M compartment. **(B)** IF staining of P-mCherry-positive particles (red) using FITC-conjugated anti-RABV antibody targeting the N protein. White arrows in merge panel indicate co-localization between P-mCherry signal and anti-N protein staining in fixed M compartment axons at 4 h post infection (scale bars = 20 μm). **(C)** RABV N protein in SCG cell bodies (CB) at 24 h post axonal infection. Protein lysates were separated using SDS-PAGE, and N protein levels were determined by western blotting. Symbols indicate the presence (+) or absence (-) of RABV infection in N.
(TIF)

S4 Fig Emetine acts locally in axons to inhibit retrograde RABV infection in a dose-dependent manner. **(A)** Quantification of % infected cell bodies at 24 h post axonal infection in the absence or presence of 10 μM, 50 μM, or 100 μM emetine in N. **(B)** Quantification of % infected cell bodies at 24 h post direct S compartment infection in the absence or presence of 100 μM emetine in N. Emetine was added to N, 1 h prior to infection in S. Emetine was washed out at 5 hpi. Black dots represent individual tri-chambers. Horizontal lines and error bars represent mean ± SD with **p = 0.004, ****p < 0.0001 using one-way ANOVA (ns = not significant using unpaired t-test).
(TIF)

S5 Fig Emetine is non-toxic when isolated axons are exposed for 6 h. **(A)** Brightfield images of cell bodies and axons at 24 h after a 6 h treatment with emetine (100 μM) or vehicle in N (scale bars = 100 μm). Emetine was washed out of the N compartment at 6 h post treatment, and fresh media was added. **(B)** Experimental setup for live/dead SYTOX cell assay: DiI was added to the N compartment axons to label connected cell bodies in red. Emetine was added to the N compartment for 6 h (or to the S compartment for 24 h as a positive control for cell death). Emetine was washed out of axons after 6 h. At 6 or 24 h, cell bodies were stained with SYTOX green nucleic acid stain (5 nM) for 10 min and imaged. Representative images show cell bodies in the S compartment at 6 h after emetine treatment in N and 24 h after emetine treatment in S (scale bars = 100 μm). **(C)** Table indicates percentage of dead cell bodies at 6 h or 24 h after a 6 h emetine treatment in N versus a 6 h or 24 h emetine treatment in S. The percentage of dead cells refers to the percentage of connected cell bodies (DiI-positive) that are stained with SYTOX (No treatment, n = 3; Emetine in N imaged at 6 h, n = 3; Emetine in N imaged at 24 h, n = 1; Emetine in S imaged at 6 h, n = 1; Emetine in S imaged at 24 h, n = 2).
(TIF)

S6 Fig RABV particles enter axons within 60 min. **(A)** Experimental setup for entry assay. **(B)** Quantification of % infected cell bodies at 24 hpi when N compartment axons were incubated with RABV inoculum for 1 to 300 minutes. The virus inoculum was removed following the designated incubation period, and the axons were washed three times with PBS to remove extracellular particles. Black dots represent individual tri-chambers. Horizontal lines and error bars represent mean ± SD with *p = 0.02, ****p < 0.0001 using one-way ANOVA (ns = not significant).
(TIF)
