# Supplementary figures and images for: Retrograde axonal transport of rabies virus is unaffected by interferon treatment but blocked by emetine locally in axons
Source: PLoS Pathog. 2018 Jul 20;14(7):e1007188. doi: 10.1371/journal.ppat.1007188 (PMC6070286; doi:10.1371/journal.ppat.1007188)

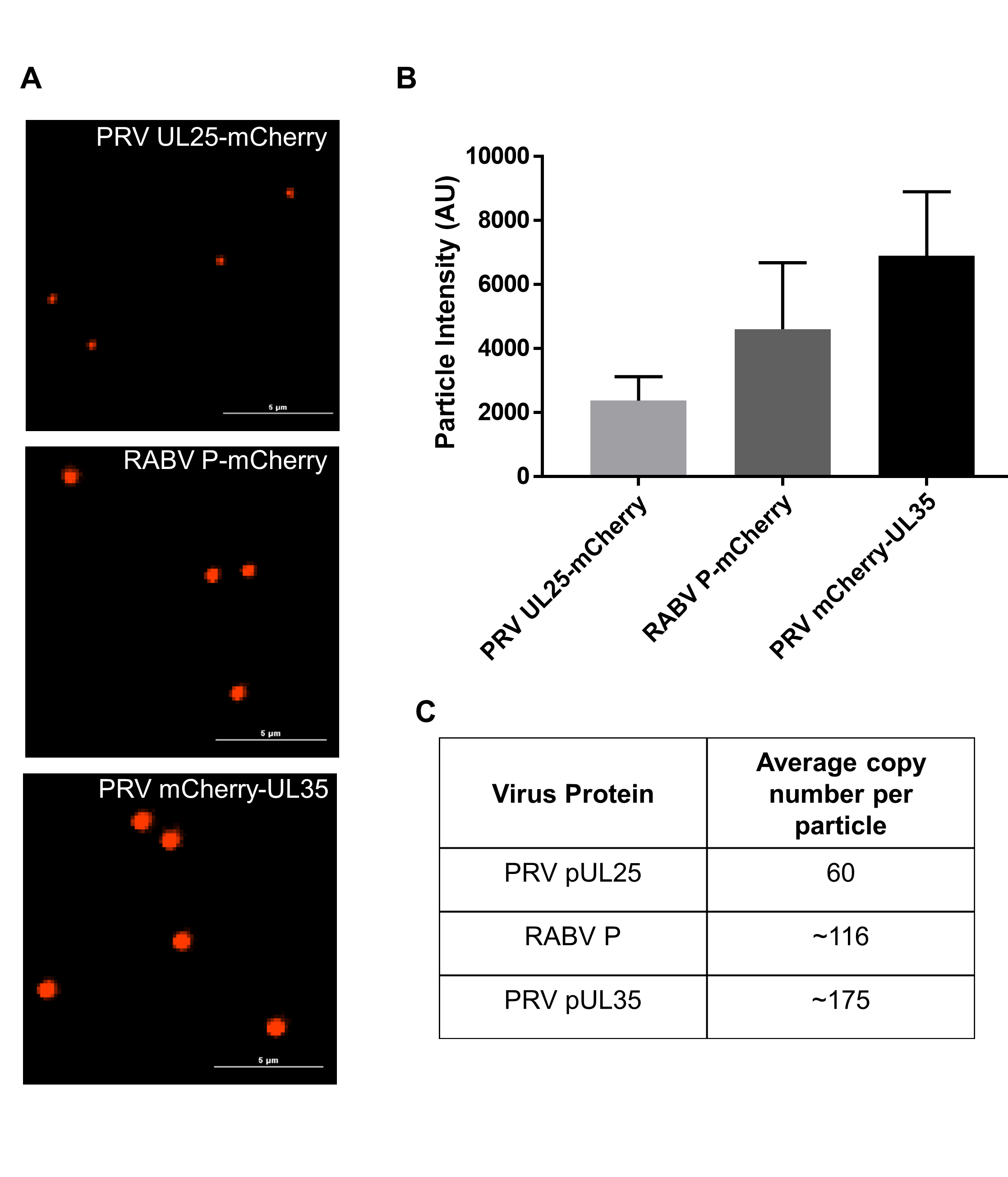

Supplement: S1 Fig — (A) PRV UL25-mCherry, RABV P-mCherry, and PRV mCherry-UL35 particles from sucrose-purified supernatants (scale bars = 5 μm). (B) Quantification of particle intensity (arbitrary units (AU)) based on mCherry brightness across individual particles of PRV UL25-mCherry (n = 815), RABV P-mCherry (n = 825), and PRV mCherry-UL35 (n = 841). Error bars indicate mean maximum particle intensity + SD. (C) Table listing the approximate average copy number per particle of RABV P and PRV pUL35. Protein copy number was extrapolated from the known PRV pUL25 copy number (60 copies) by comparing the mCherry fluorescence emission intensities between virus strains. (TIF) [file ppat.1007188.s001.tif]

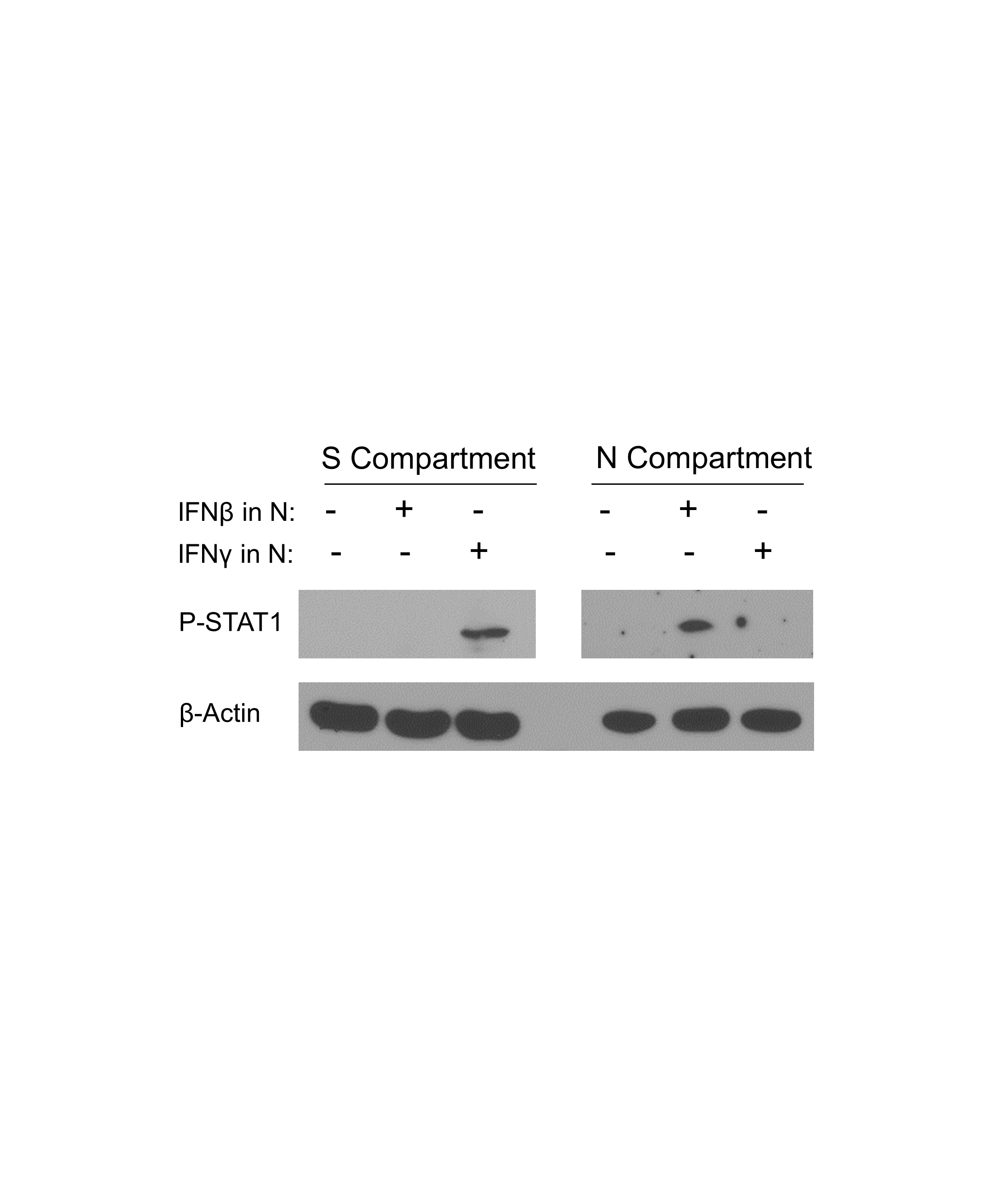

Supplement: S2 Fig — N compartment axons were treated with IFNβ or IFNγ for 24 h. S and N compartments were lysed separately, and proteins were separated by SDS-PAGE on 4–12% gradient gels. Phosphorylated STAT1 (P-STAT1) levels were determined in the S and N compartments by western blotting. Symbols indicate the presence (+) or absence (-) of IFN in the N compartment. Β-actin served as a loading control. (TIF) [file ppat.1007188.s002.tif]

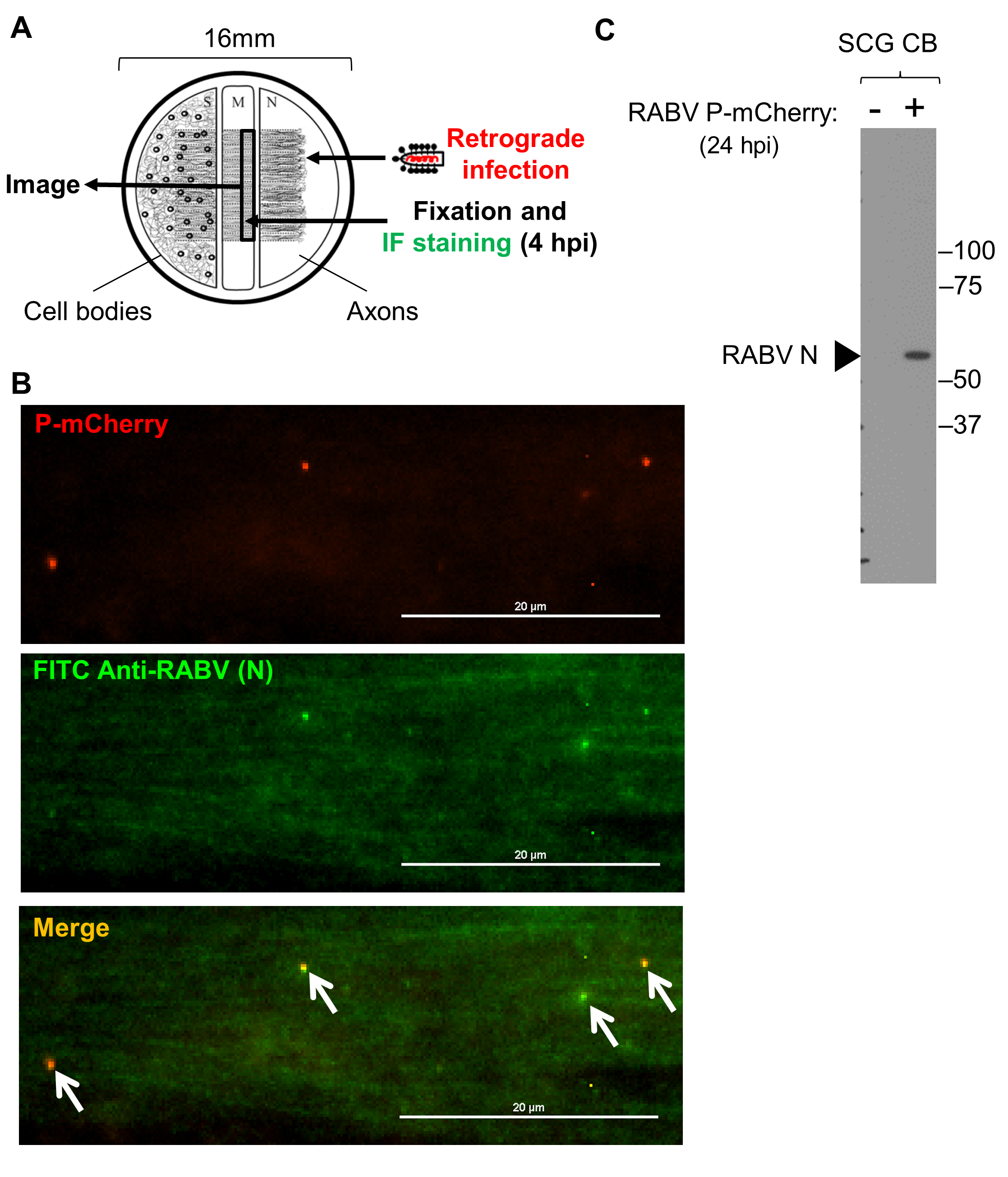

Supplement: S3 Fig — (A) Experimental setup for immunofluorescence (IF) staining of RABV particles in the M compartment. (B) IF staining of P-mCherry-positive particles (red) using FITC-conjugated anti-RABV antibody targeting the N protein. White arrows in merge panel indicate co-localization between P-mCherry signal and anti-N protein staining in fixed M compartment axons at 4 h post infection (scale bars = 20 μm). (C) RABV N protein in SCG cell bodies (CB) at 24 h post axonal infection. Protein lysates were separated using SDS-PAGE, and N protein levels were determined by western blotting. Symbols indicate the presence (+) or absence (-) of RABV infection in N. (TIF) [file ppat.1007188.s003.tif]

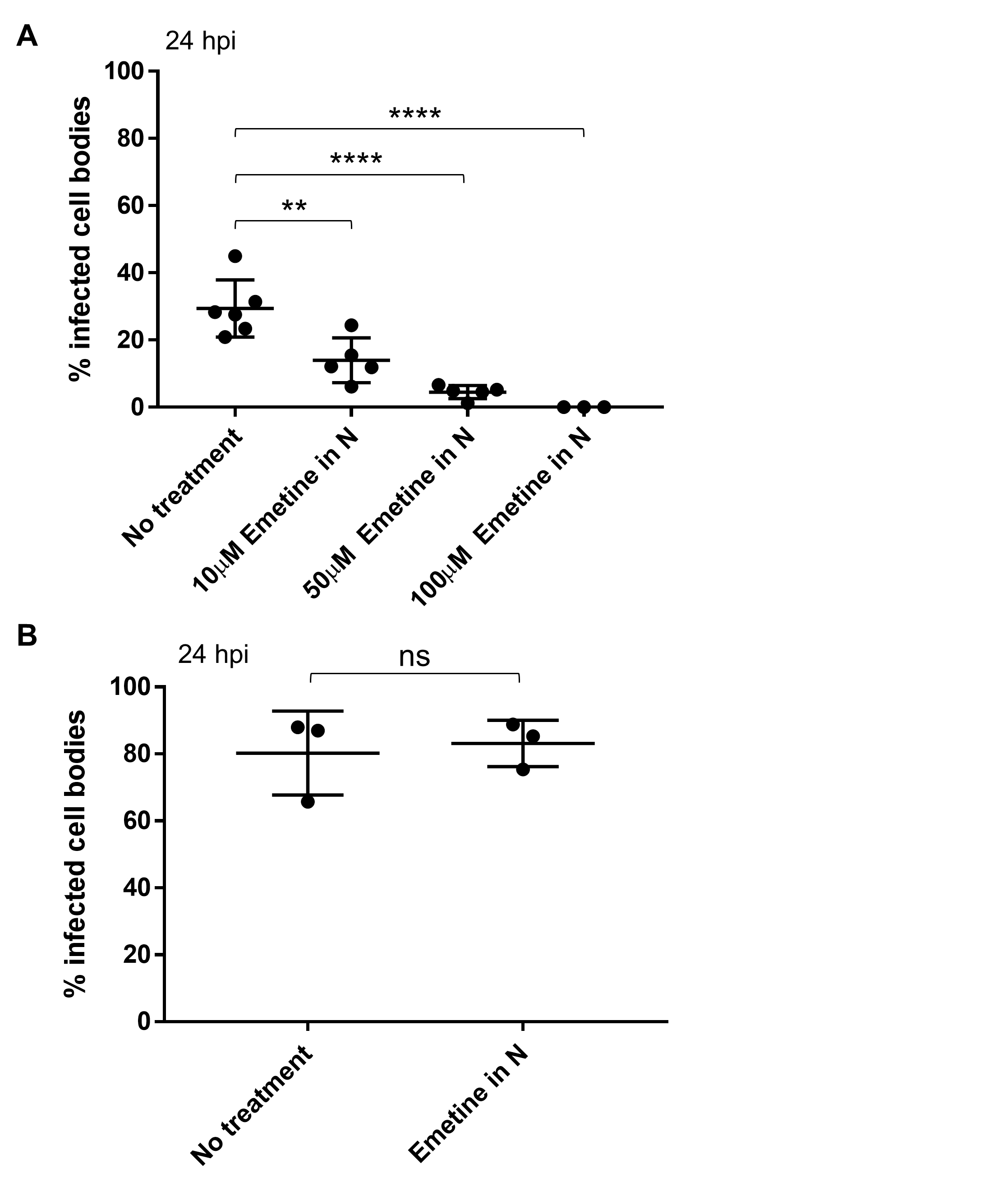

Supplement: S4 Fig — (A) Quantification of % infected cell bodies at 24 h post axonal infection in the absence or presence of 10 μM, 50 μM, or 100 μM emetine in N. (B) Quantification of % infected cell bodies at 24 h post direct S compartment infection in the absence or presence of 100 μM emetine in N. Emetine was added to N, 1 h prior to infection in S. Emetine was washed out at 5 hpi. Black dots represent individual tri-chambers. Horizontal lines and error bars represent mean ± SD with **p = 0.004, ****p < 0.0001 using one-way ANOVA (ns = not significant using unpaired t-test). (TIF) [file ppat.1007188.s004.tif]

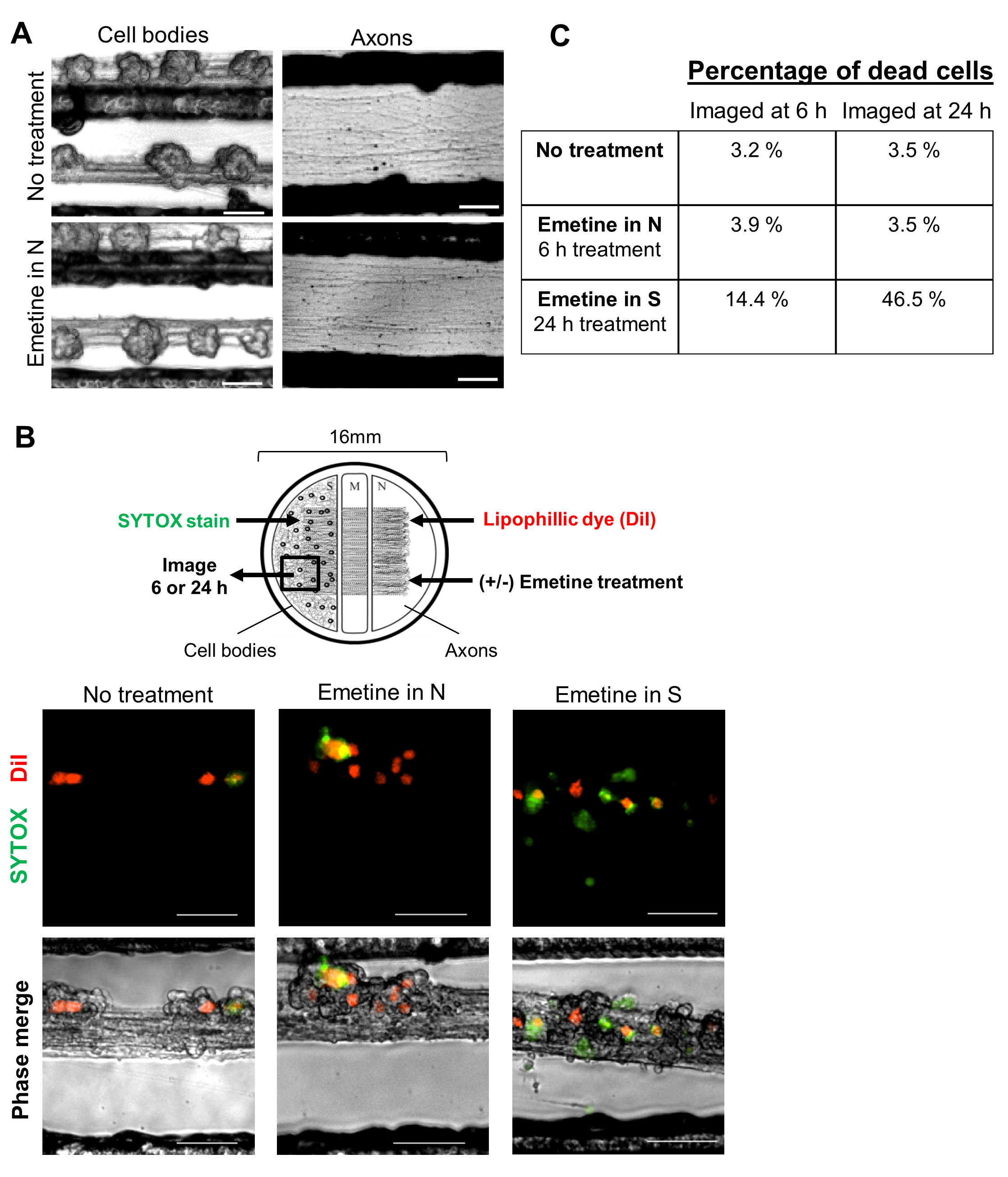

Supplement: S5 Fig — (A) Brightfield images of cell bodies and axons at 24 h after a 6 h treatment with emetine (100 μM) or vehicle in N (scale bars = 100 μm). Emetine was washed out of the N compartment at 6 h post treatment, and fresh media was added. (B) Experimental setup for live/dead SYTOX cell assay: DiI was added to the N compartment axons to label connected cell bodies in red. Emetine was added to the N compartment for 6 h (or to the S compartment for 24 h as a positive control for cell death). Emetine was washed out of axons after 6 h. At 6 or 24 h, cell bodies were stained with SYTOX green nucleic acid stain (5 nM) for 10 min and imaged. Representative images show cell bodies in the S compartment at 6 h after emetine treatment in N and 24 h after emetine treatment in S (scale bars = 100 μm). (C) Table indicates percentage of dead cell bodies at 6 h or 24 h after a 6 h emetine treatment in N versus a 6 h or 24 h emetine treatment in S. The percentage of dead cells refers to the percentage of connected cell bodies (DiI-positive) that are stained with SYTOX (No treatment, n = 3; Emetine in N imaged at 6 h, n = 3; Emetine in N imaged at 24 h, n = 1; Emetine in S imaged at 6 h, n = 1; Emetine in S imaged at 24 h, n = 2). (TIF) [file ppat.1007188.s005.tif]

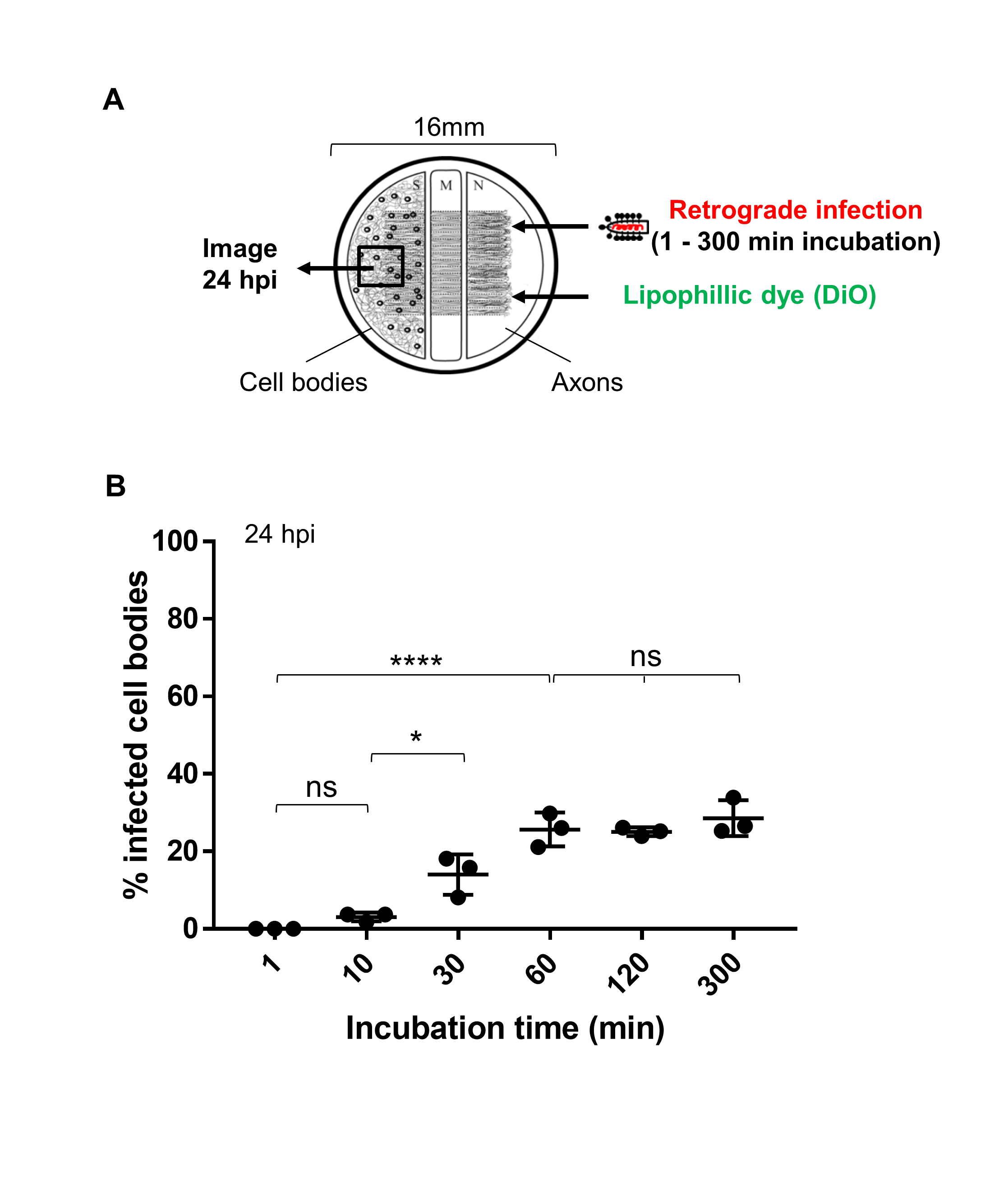

Supplement: S6 Fig — (A) Experimental setup for entry assay. (B) Quantification of % infected cell bodies at 24 hpi when N compartment axons were incubated with RABV inoculum for 1 to 300 minutes. The virus inoculum was removed following the designated incubation period, and the axons were washed three times with PBS to remove extracellular particles. Black dots represent individual tri-chambers. Horizontal lines and error bars represent mean ± SD with *p = 0.02, ****p < 0.0001 using one-way ANOVA (ns = not significant). (TIF) [file ppat.1007188.s006.tif]
